# Magnetohydrodynamic-based Internal Cooling System for a Ceramic Cutting Tool: Concept Design, Numerical Study, and Experimental Validation

**DOI:** 10.1007/s41871-023-00210-9

**Published:** 2023-08-29

**Authors:** John O’Hara, Fengzhou Fang

**Affiliations:** 1 Center of Micro/Nano Manufacturing Technology (MNMT-Dublin), University College Dublin, Dublin 4, Ireland; 2 State Key Laboratory of Precision Measuring Technology and Instruments, Laboratory of Micro/Nano Manufacturing Technology (MNMT), Tianjin University, Tianjin, 300072 China

**Keywords:** Internal cooling, Heat transfer, Liquid metal, Magnetic field, Cutting tool

## Abstract

The effective removal of the heat generated during mechanical cutting processes is crucial to enhancing tool life and producing workpieces with superior surface finish. The internal cooling systems used in cutting inserts employ a liquid water-based solvent as the primary medium to transport the excess thermal energy generated during the cutting process. The limitations of this approach are the low thermal conductivity of water and the need for a mechanical input to circulate the coolant around the inner chamber of the cutting tool. In this context, this paper proposes an alternative method in which liquid gallium is used as the coolant in combination with a magnetohydrodynamic (MHD) pump, which avoids the need for an external power source. Using computational fluid dynamics, we created a numerical model of an internal cooling system and then solved it under conditions in which a magnetic field was applied to the liquid metal. This was followed by a simulation study performed to evaluate the effectiveness of liquid gallium over liquid water. The results of experiments conducted under non-cooling and liquid gallium cooling conditions were analyzed and compared in terms of the tool wear rate. The results showed that after six machining cycles at a cutting speed *V*_*c*_ = 250 m min ^−1^, the corner wear VB_c_ rate was 75 µm with the coolant off and 48 µm with the MHD-based coolant on, representing a decrease of 36% in tool wear. At *V*_*c*_ = 900 m min^−1^, the corner wear VB_c_ rate was 75 µm with the coolant off and 246 µm with the MHD-based coolant on, representing a decrease of 31% in tool wear. When external cooling using liquid water was added, the results showed at *V*_*c*_ = 250 m min^−1^, the difference between the tool wear rate reduction with the internal liquid gallium coolant relative to the external coolant was 29%. When the cutting speed was increased to *V*_*c*_ = 900 m min^−1^, the difference observed between the internal liquid gallium coolant relative to the external coolant was 16%. The study proves the feasibility of using liquid gallium as a coolant to effectively remove thermal energy through internally fabricated cooling channels in cutting inserts.

## Highlights

1. Numerical modeling data indicates that liquid gallium is a superior heat transfer substance compared to liquid water for use in cutting inserts with geometrically specified internal dimensions.
2. Machining tests show that the insert with MHD cooling system outperformed in terms of heat transfer, compared to the inserts without any coolant under the same conditions. This was also observed when compared to external coolants using liquid water. Furthermore, the elemental metal is compatible with oxide ceramics within an additively manufactured internal cooling channel.
3. The results indicate that as the cutting speed increases, the liquid gallium provides increased heat transfer capabilities. This suggests a correlation between the increasing heat and the efficiency of liquid gallium as a thermal transfer agent.

## Introduction

### Need for Heat Reduction in Mechanical Cutting Processes

Heat reduction in micro- and mesoscale engineering systems is critical to maintaining and developing sustainable solutions to current challenges in thermal management [1]. Micro-manufacturing operations produce heat as a by-product of machine use [2]. In precision machining applications, the excess tool wear due to the heat induced during cutting actions is a major challenge in thermal management [3, 4]. The necessity for a cooling mechanism in machining operations has been well documented [4–6]. Cooling, either by external or internal approaches, helps reduce tool wear and improve thermal degradation, which affects the surface integrity of workpieces [6, 7]. Currently, internal cooling within the tool structure is gaining interest as an alternative to conventional thermal flooding methods, which are expensive, potentially hazardous, and harmful to the environment [8–10].

The unique thermophysical properties of ceramic materials make them ideal for applications requiring high heat resistance [11]. Aluminum oxide (Al_2_O_3_) is a chemically stable oxide of trivalent aluminum [12]. As a polycrystalline material, it is classified as a technical ceramic and is widely used in applications requiring high hot hardness, high wear resistance, good thermal insulation, and chemical inertness [12]. Due to their brittle nature, Al_2_O_3_ cutting inserts are prone to chipping, and therefore, continuous uninterrupted cutting regimes are a prerequisite for successful use. In this study, we considered only high-purity aluminum oxide powders (99.9%), which are representative of the ceramic slurry used in the forming process (see Table 1).

**Table 1:** Physical properties of sintered LithaLox 500 alumina (Al_2_O_3_) [13]

As-sintered sample	Value
Relative density [%]	98.4
Porosity [%]	1.6
Purity [%]	99.8
Surface roughness Ra [μm]	0.9
Theoretical density [g/cm^3^]	3.985
Hardness [HV10]	1450
Thermal conductivity [W/(m∙K)]	37
Maximum operating temperature [°C]	1650
Specific electrical resistivity [Ω∙cm]	~ 1014
Relative permittivity	9.8–10
Young’s modulus [GPa]	300
Fracture toughness [MPa∙m^1/2^]	4–5
Four-point bending strength [MPa]	430

Technical ceramics perform effectively when used as a cutting tool on cast iron, superalloys, and hardened steels [14, 15]. The ability to withstand high cutting temperatures without degradation allows ceramics to perform optimally under high-speed cutting conditions [11]. In fact, the high temperatures generated in the cutting zone are conducive to a ceramic cutting tool because the removal of material using ceramic tools does not proceed in the same manner as that in the case of metallic cutting tools [11, 16].

However, like all materials, ceramics eventually degrade, and the high temperatures and cutting conditions result in tool wear. Coolant supply can help enhance the tool life when using metallic cutting tools. However, this is not observed when using ceramic tools, which perform better under dry machining conditions [11, 17]. To provide an alternative heat transfer method to the use of external coolants for cutting inserts, an integrated internal cooling system is well suited for ceramic materials. In this study, the general flexibility of additive manufacturing was utilized for the creation of novel features in ceramic materials, which form the basis of this work in terms of the fabrication of internal channels.

The use of an internal coolant technology in cutting tools is associated with two limitations: (1) the low heat transfer capacity of the liquid water and (2) the requirement for a mechanical input to pump the coolant around the internal channel, thus demanding a constant external power source. Hence, this paper proposes an alternative approach to overcome these limitations.

### Liquid Gallium as a Heat Transfer Agent

Owing to its excellent thermal properties, liquid gallium and its alloys have been applied as coolants based on magnetohydrodynamic (MHD) heat transfer mechanisms [18–21]. To transport heat at smaller scales, liquid gallium outperforms other liquid metals, such as mercury, where higher vaporization temperatures and nontoxic characteristics are required [18]. Al Omari [22] compared liquid gallium and liquid mercury as cooling agents in transferring thermal energy from water and demonstrated that liquid gallium outperformed liquid mercury. Sarowar [23] used a series of liquid gallium alloys for thermal transport in ceramic micro-heat sink substrates. Each alloy performed differently in terms of the heat transfer efficiency, but overall, the cooling in the microchannels was improved. The author emphasized the role of ceramics in compatible substrate materials with liquid gallium alloys. Xu et al. [24] compared a liquid gallium heat sink and a copper/paraffin alternative in terms of their heat transfer efficiency. Liquid gallium performed better overall, and its effective thermal conductivity was significantly higher (71.97 W/(m·K)) for steady-state heating than that of solid gallium, thus enabling superior thermal management control. The authors suggested that the natural convection during melting contributes to enhancing the quantity of heat transfer. Further applications merge liquid gallium with energy storage potential and thermal transfer. This was demonstrated by Salyan et al. [25], where liquid metal was used as part of an energy storage/heat transfer mechanism in metal inserts within a solar thermal storage system. Kim et al. [26] highlighted the potential of liquid metals, including gallium, as deformable sensors and actuators in electronics. Another advantage of using liquid metal-based magnetohydrodynamic systems for heat exchangers is the ease in which permanent magnets can be applied to generate a magnetic field; these magnets can operate at lower temperatures, thus reducing the demagnetization potential [27].

### Elemental Gallium: Properties

Liquid gallium has an excellent thermal conductivity of ~ 40 Wm^−1^ K^−1^ at 0.01 MPa (450 K) and is nontoxic in its elemental form. These properties enable it to transfer heat in channels with dimensions at the microscale with relatively low flow rates, requiring good thermal conductivity [28, 29]. It has a high boiling point of 1983 °C with a density of 6 g cm^−3^ at 33 °C [28]. It is electrically conductive and paramagnetic. Table 2 presents the properties of elemental gallium*.* Liquid gallium undergoes rapid oxidation in the presence of air [28], producing a thin viscous film on its surface. It has one of the highest thermal conductivities among metals, reaching up to ~ 79.1 Wm^−1^ K^−1^ in its liquid form [24]. Liquid gallium, when exposed to temperatures above 350 °C, can be corrosive to certain metallic alloys such as stainless steel [30, 31]; this corrosive effect is negligible at lower temperatures.

**Table 2:** Physical properties of elemental gallium [32]

Parameter	Magnitude
Density (at 20 °C)	5900 kg/m^3^
Melting point	29.78 °C
Boiling point	2300 °C
Latent heat of melting	8.03 × 10^4^ J/kg
Coefficient of linear expansion (at 20 °C)	1.8 × 10^5^ °C^−1^
Specific heat (in the range of 0–16 °C)	0.373 × 10^3^ J/kg K
Thermal conductivity	29.308–37.681 W/m·K
Electrical resistivity	4.5 × 10^−7^ Ω·m
Temperature coefficient of electric resistance (in the range of 0–16 °C)	3.96 × 10^3^ °C^−1^
Brinell hardness	2.5 × 10^6^ kg/m^2^
Compressibility coefficient (at 20 °C)	2 × 10^−10^ m^3^/kg
Tensile strength	(2.0–3.8) × 10^6^ kg/m^2^
Elongation per unit length	2%–40%
Evaporation pressure	10^−12^ mm/Hg
Surface tension	0.707 N m^−1^

Gallium is a solid metal at room temperature, and it shrinks in volume when phase transitioned from solid to liquid state with a corresponding change in its structure, reflecting a shrinkage of 5.3 × 10^−6^ m^3^/kg at 29.8 °C [32]. When in the liquid phase, its electrical conductivity increases along with its coordination number. An unusual property of its molten state is that it can remain in a super-cooled liquid phase, before solidification re-occurs, for relatively long periods (several weeks) if not stirred [32]. Gallium does not chemically react with most ceramics including aluminum oxide and hexagonal boron nitride (hBN). These properties indicate that liquid gallium can be a potential alternative to liquid water for use in the proposed design as a heat transfer fluid within a ceramic cutting insert.

## Fundamentals of Magnetohydrodynamics

MHD is a model of the electrodynamic phenomena of conducting liquids under the action of an applied magnetic field [19]. Therefore, it requires a combined approach involving electromagnetic theory and fluid mechanics. In this study, MHD is developed where the generation of the steady-state model is derived from Maxwell’s equations for electromagnetic fields coupled with the Navier–Stokes equations for fluid dynamics. The physical laws describing the interaction between the magnetic field and the electrically conducting fluid are derived through the combination of Maxwell’s laws of electromagnetism and Navier–Stokes equations dealing with the conservations of mass and momentum. The MHD theory involves the coupling of the velocity field *µ* and the magnetic field *B*_0_, from which the dynamic behavior of the circulating fluid (in this case) produces an electric current *J* (also called the eddy current), within the fluid body in the presence of the applied magnetic field, which is governed by Ohm’s law:

2.1 $$J=\sigma \left(E+\mu \times B\right)$$

where *σ* is the electric conductivity of the fluid, *E* is the induced electric field, *µ* is the velocity field, and *B* is the magnetic field. This equation thus defines the electric current density. In the case under consideration, the electric field *E*, within the fluid, is generated because of the kinematic motion of the conducting liquid metal interacting with the magnetic field lines. This is a direct consequence of Faraday’s law of induction:

2.2 $$\nabla \times E=-\frac{\partial B}{\partial t}$$

where ∇ is the curl operator, *E* is the electric field, and *B* is the magnetic field. The coupling between the velocity field *µ* and the magnetic field *B*_0_ also produces an electromotive force in liquid gallium, known as the Lorentz force, given by

2.3 $$F=\left(J\times B\right)$$

Considering the fluid dynamics of the conducting liquid metal, this electromotive force is introduced as an additional force in the Navier–Stokes equations, which in turn changes the flow dynamics, and the resulting equation is based on the conservation of mass; however, as in this case, it is incompressible and therefore simplified to the continuity equation,

2.4 $$\nabla \cdot \mu =0$$

Here, a steady-state laminar flow is assumed, which is incompressible (the volumetric strain rate is zero), for liquid gallium flowing under the influence of a uniform magnetic field. Additionally, the fluid behavior is Newtonian with a relatively low Reynolds number. The second term relating to the fluid dynamics of the system is the momentum equation, given by

2.5 $$\frac{\text{d}u}{\text{d}t}+\left(u\cdot \nabla \right)u=-\frac{1}{\rho \sigma }\nabla P+\mu {\nabla }^{2}u+\frac{1}{\rho }+F$$

where $u$, $\rho$, *µ*, *P*, and *σ* are the velocity, density, kinematic viscosity, pressure, and electric conductivity, respectively. In the developed model, the fluidic properties, density $\rho$, electric conductivity *σ*, and kinematic viscosity *µ* are considered constants. From Eq. (2.5), it can be seen that the electromotive or Lorentz force (represented by *F*) is the driving force for fluid flow within the system. Furthermore, based on the assumptions made regarding the flow behavior (laminar, incompressible, and steady), a no-slip assumption is made on the wall velocities along the *y* and *z* directions (being zero), with surface tension ignored. Accordingly, Eqs. (2.4) and (2.5) can be further simplified according to the conservation of mass as

2.6 $$\frac{\text{d}u}{\text{d}x}=0$$

and according to the conservation of momentum as follows:

2.7 $$0= \frac{\partial p}{\partial x}+\mu \left(\frac{{\partial }^{2}u}{{\partial y}^{2}}+\frac{{\partial }^{2}u}{{\partial z}^{2}}\right)$$

**Fig. 1:**
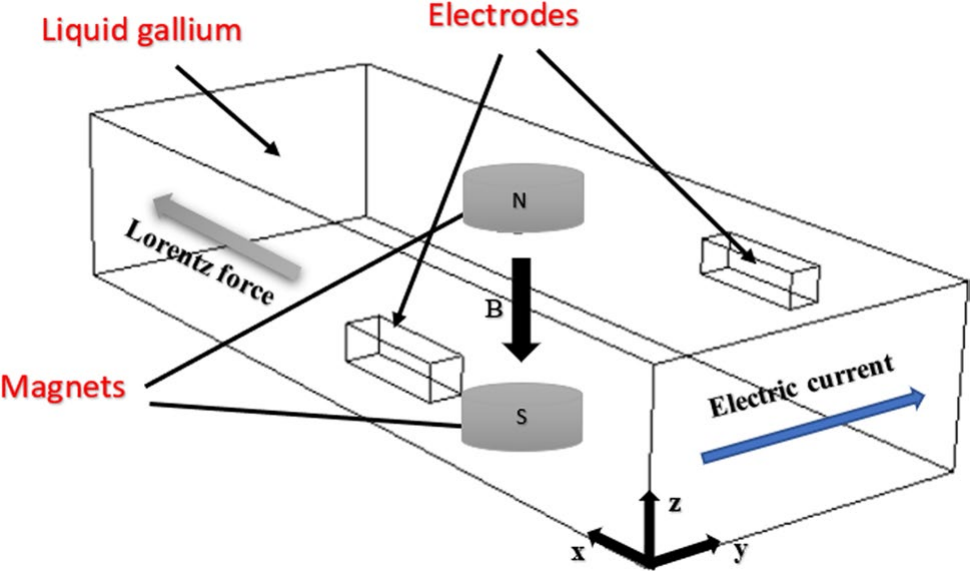
Schematic of a section of the internal channel region in an Al_2_O_3_ insert, illustrating the mechanism of fluid dynamics resulting from the external magnetic field and fixed electrodes

**Fig. 2:**
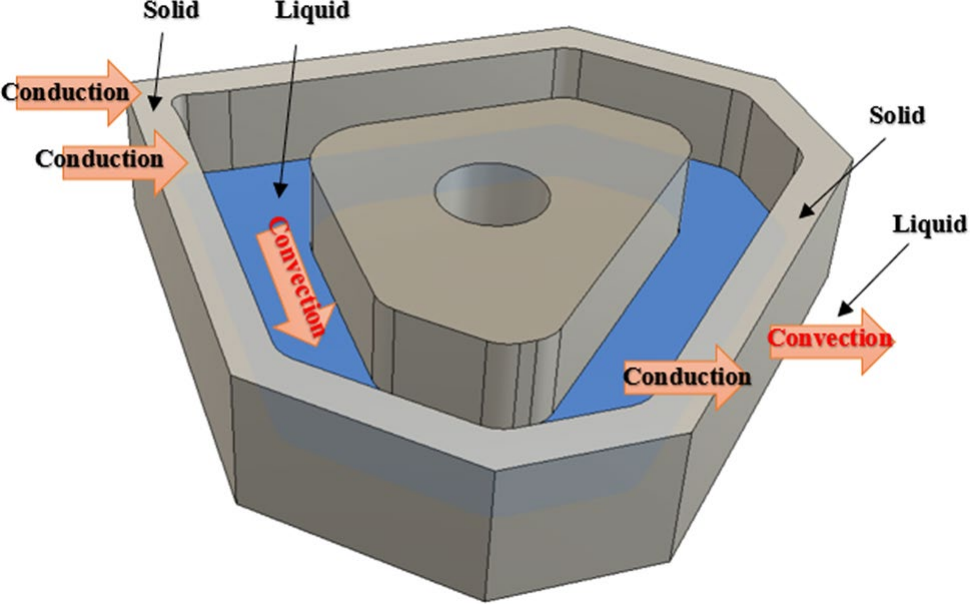
Model of the internal heat transfer route through the walls of a solid aluminum oxide insert, through the liquid metal, and then out through the solid wall of the insert

The scenario is therefore a 3D liquid metal MHD flow problem within the internal channel of a customized Al_2_O_3_ insert shown in Fig. 1. Figure 2 shows a cut-away section of the CAD model of the internal channel within the Al_2_O_3_ insert, demonstrating the heat transfer mechanism.

Figure 1 shows a schematic of a closed channel containing liquid gallium with two electrodes fixed against the outer chamber walls*.* When a potential difference is applied across the fixed electrodes, a flux $J$ flows in the liquid. A magnetic field $B$, generated from the neodymium permanent magnets, interacts with the flux $J$, creating a Lorentz force $J\times B.$ This results in a fluidic motion within the closed channel system. The MHD drive produces a circulating thermal current, which is cyclically driven across the phase boundaries toward the heat sink, as illustrated in Fig. 2*.* MHD propulsion does not require external moving parts and, along with the excellent thermal conductivity and high boiling point of gallium (Table 1), offers a novel solution for the internal cooling of ceramic cutting inserts.

The underlying principle of the fluid flow driven by an MHD pump is based on the interaction between the conducting fluid (liquid gallium), electric current, and the applied magnetic field *B*, whereby a force is generated (Ampère’s force), which drives the liquid metal around the borders of its confined dimensional space. This force is perpendicular to the magnetic field* B* and the flow direction of the conducting liquid.

The interest in the use of liquid metal as an enhanced thermal transfer agent is owing to its high heat transfer coefficient, low melting point, high boiling point at normal pressure, low toxicity (compared to mercury), and reusability.

## Challenges in Thermal Management

### Origin of the Problem

**Fig. 3:**
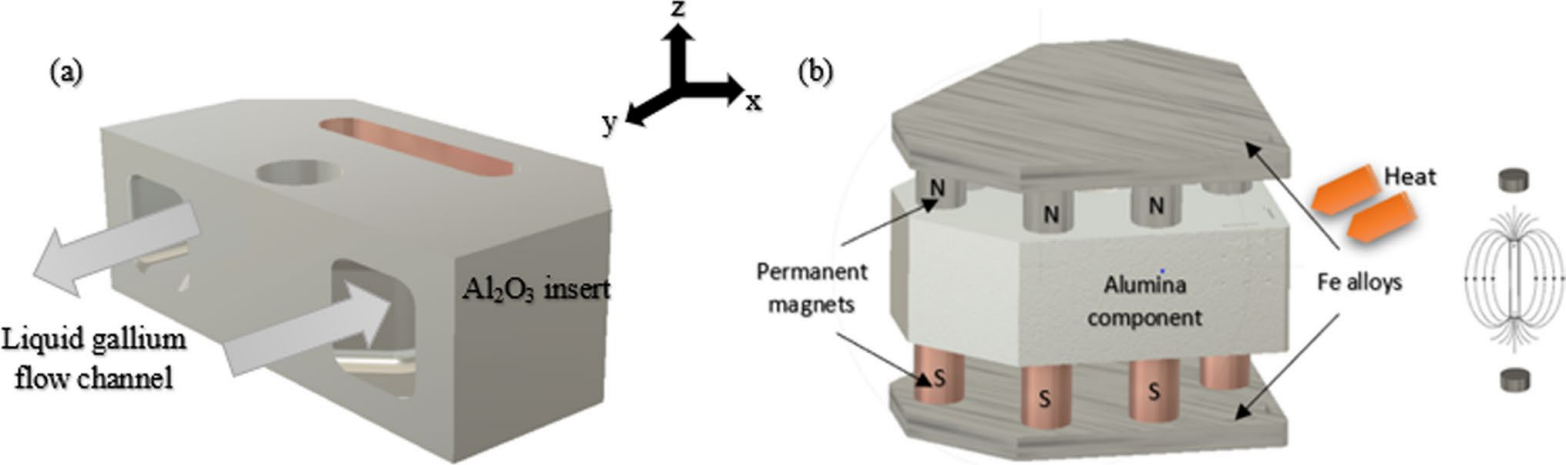
**a** Model of the internal channels in the insert and location of liquid gallium. **b** Illustration of the mechanism: Permanent magnets with opposite polarity are aligned perpendicular to the flow direction of internally contained liquid metal (Note: Number of magnets exaggerated for illustration)

**Fig. 4:**
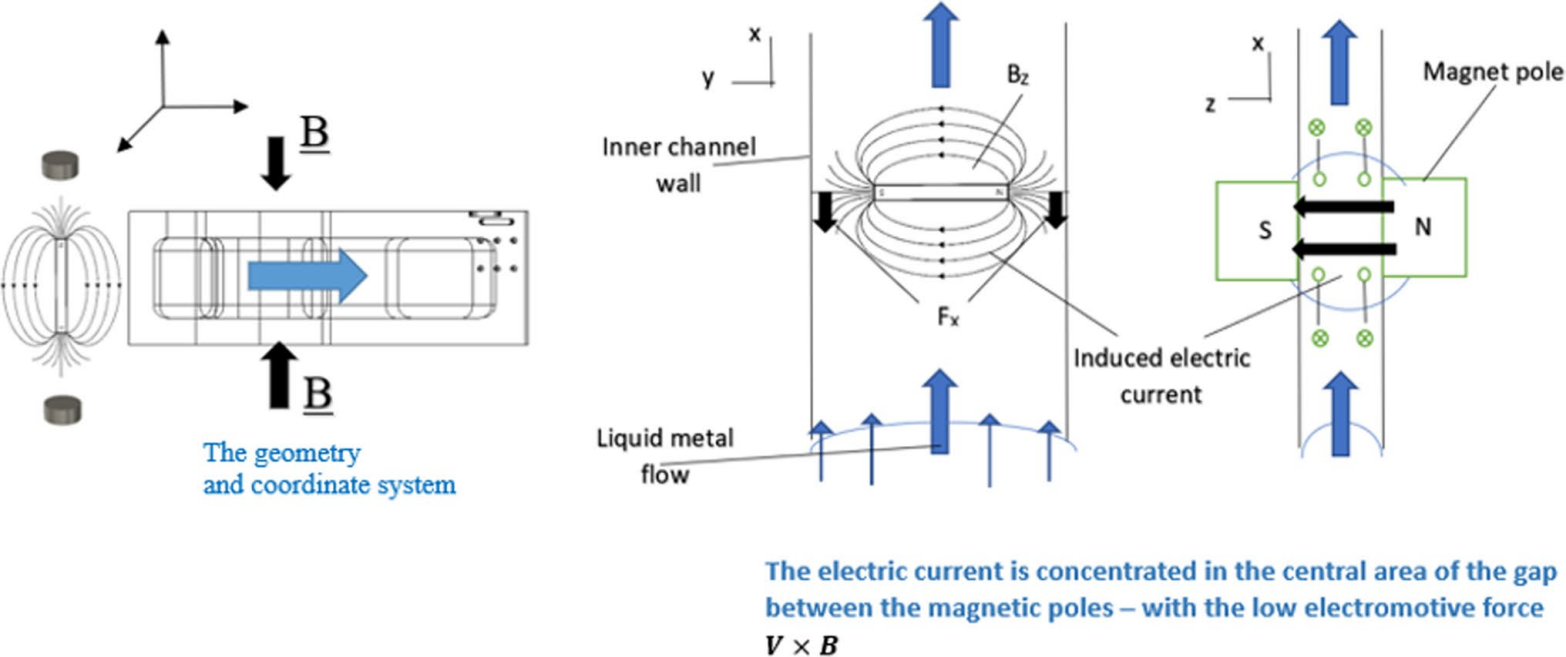
Schematic of the mechanism of action of the applied magnetic field B on a liquid metal flowing in the channel of an aluminum oxide insert

The problem addressed in this study can be described as the flow of a steady-state, laminar, viscous, electrically conducting liquid metal in a fixed geometrical region of the internal channels within the Al_2_O_3_ cutting insert, as shown in Fig. 3a*.* A series of neodymium permanent magnets generate a homogeneous magnetic field perpendicular to the liquid metal within the channel, as shown in Fig. 3b. The behavior of the liquid metal (elemental gallium: 99.8%, *Goodfellow*) was analyzed under the influence of six fixed permanent neodymium magnets (*Eclipse*) with a diameter *d* = 3 mm and height *h* = 2 mm, located at opposite ends of the additively manufactured aluminum oxide insert. The magnetic field on the surface of the insert is ± *B*_*o*_*e*_*Z*_ with a north–south polarity arranged according to the geometrical setup shown in Fig. 3b. When an electric current is applied to the liquid metal via the electrodes, the liquid gallium (now behaving as a laminar fluid) and the induced kinetic motion help maintain the liquid state. This can be understood in terms of the magnetic field B applied perpendicular to the flow direction of liquid gallium (Fig. 4*)*. The liquid metal is assumed to be electrically conducting, incompressible, and in a steady state within the cylindrically shaped channel. The magnetic field on the surface of the insert is ± *B*_*o*_*e*_*Z*_ with a magnitude of ~ 1.2 T (the magnetic field strength of the neodymium magnets)*.* Using the value *B*_*o*_ to represent the applied magnetic field, the magnitude can be given by:

3.1 $${Ha}= {B}_{o}H\sqrt{\left(\frac{\sigma }{\rho v}\right)}$$

where *Ha* is the dimensionless Hartmann number, which is the ratio of the electromagnetic forces to the viscous forces, $\sigma$ is the electric conductivity, *ρ* is the density, and *v* is the velocity. Regarding the liquid metal, it is assumed to flow as an incompressible fluid at a velocity *U*_*o*_, and applying the ratio of inertial and viscous flow yields:

3.2 $${Re}=\frac{{U}_{o}H}{v}$$

here *Re* is the dimensionless Reynolds number, and *v* is the kinematic viscosity. The Reynolds number represents the laminar flow behavior within the MHD system, producing stable flow currents along the wall boundaries of the internal channel. The walls of the Al_2_O_3_ insert are electrically insulating; therefore, the solid domain for this problem is zero. Finally, by applying the geometric parameters defined for the problem, along with the values for *Re*, *Ha*, and the physical properties of elemental gallium (Table 1), we can determine the boundary conditions for the model. From a thermal perspective, liquid metals differ from other liquid media (e.g., water) due to their higher thermal conductivity *λ* (W/Mk) and lower specific heat capacity *c*_*p*_ (J/kg·K) [26]. The two parameters are typically combined into a dimensionless number, called the Prandtl number (*Pr*), applied to heat transfer problems in fluid dynamics. This dimensionless number represents the ratio of momentum diffusion to thermal transport in a fluid, given by the formula:

3.3 $${Pr}=\frac{{\rho}v{c}_{p}}{\lambda }=\frac{v}{k}, k= \frac{\lambda }{{\rho}{c}_{p}}$$

where *c*_*p*_ is the specific heat capacity, *ρ* is the density, *λ* is the thermal conductivity, and *k* is the thermal diffusivity. Liquid metals, such as gallium, have a significantly lower Prandtl number than media, such as water, with the former being ≈ 0.028.

Figure 4 shows the mechanism of action whereby a homogeneous magnetic field is generated. The magnetic field acts on the liquid metal, causing a dynamic effect that results in a constant cyclic flow around the closed channel. Heat is first transferred via conduction into the solid insert, then across the boundary, and finally into the convection zone.

### Relevance of the Challenges Associated with Heat Transfer

To integrate an MHD-based cooling system into an additively manufactured ceramic insert, a novel approach was applied to solve the challenges associated with the heat transfer in mechanical cutting processes. Fluid circulation through mechanical pumping invariably requires an external power source. This in turn requires a fluid reservoir that can replenish the cooling fluid once it completes a cycle in the internal channel. To avoid these requirements, the application of a magnetic field combined with the use of liquid gallium as the coolant replaces the conventional mechanical pumping system. Therefore, the challenge is to use Maxwell’s equations for the electromagnetic fields coupled with Navier–Stokes equations to numerically model the dynamics of the problem, for which a physical model was built, tested, and compared. If feasible, the solution may offer a superior method for heat transfer through the enhanced thermal conductivity of liquid gallium and without requiring mechanical power in the form of a pump.

## Materials and Methods

### Requirements and Application

**Fig. 5:**
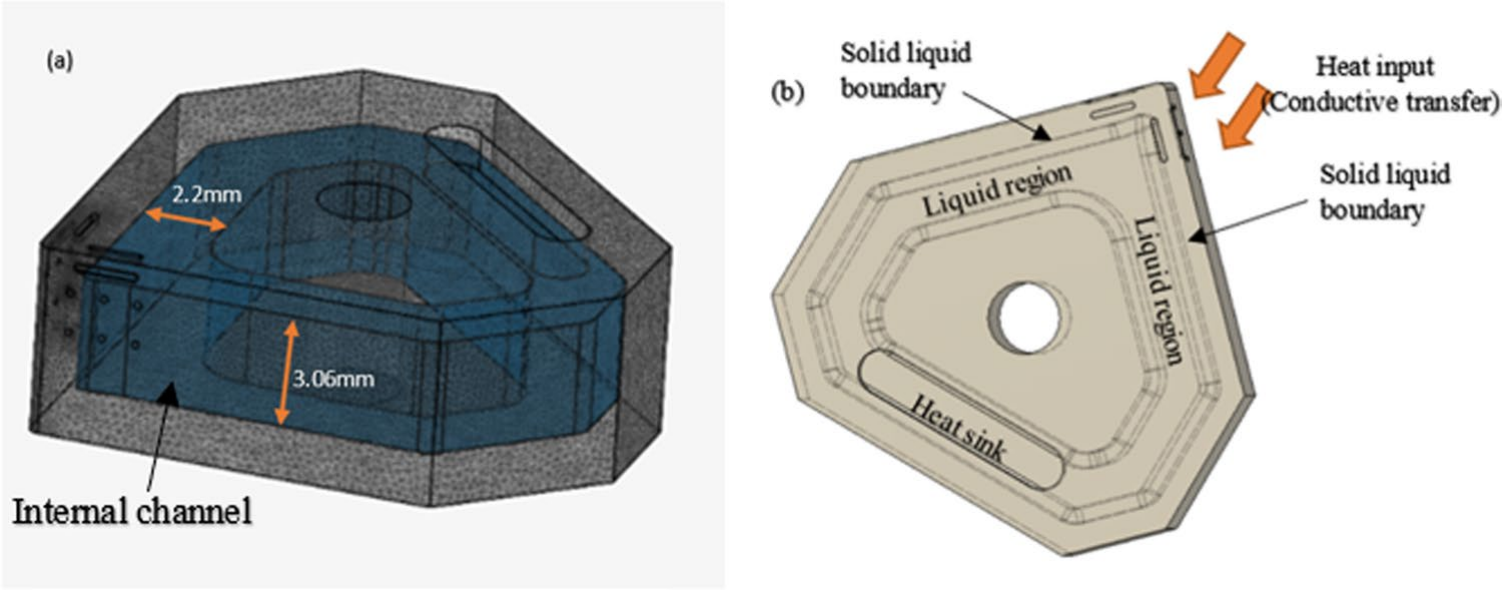
**a** Model of the internal channel with dimensions. **b** Planar view of the Al_2_O_3_ insert highlighting the solid–liquid boundary, liquid partition, and heat sink

Although technical ceramics, such as alumina and zirconia, are used without coolants, tool wear still occurs during machining, and the excess heat generated can adversely affect tool life and surface finish of the workpiece [14]. The delivery of the coolant to the ceramic tool necessitates a specific form of heat removal because ceramic materials are prone to thermal shock, which can result in fracture and failure during use [14]. Therefore, a gradual reduction in heat rather than rapid quenching is required to avoid fractures induced by thermal shock, which is produced in the case of external flooding. Internal cooling can successfully remove the heat generated at the cutting tip, thereby extending the tool life and improving surface finish [6–9]. Generally, liquid water is used as a coolant and driven cyclically around the internal channel within the insert by means of a mechanical pump. Figure 5a shows an illustration of the internal channel dimensions with the corresponding phase boundaries and heat sink shown in Fig. 5b*.*

### Fabrication

The ceramic inserts were fabricated using a high-purity aluminum oxide (99.99%) photocurable ceramic suspension (LithaLox HP 500). This required a ceramic-based powder blend and a photocurable polymer matrix suspension. With the use of a 3D lithographic ceramic manufacturing (LCM) printer (CeraFab 7500) and a standard tessellation language (STL) data format, the model can be transferred to a digital platform for relative positioning as required. The software provides a modification function for geometrical dimensioning to account for shrinkage during the densification phase. In the process used in this study, a scaling factor of 1.245 was applied for the imported STL files to compensate for the shrinkage during sintering. Additional processing parameters (including the layer thickness, light intensity, and exposure time) are part of the forming phase whereby the LCM software provides optimized outputs in terms of the rheology and dispersion rates of the photocurable matrix, thus affecting the overall quality of the ceramic green body [33]. In this study, a layer thickness of 25 µm was used.

**Fig. 6:**
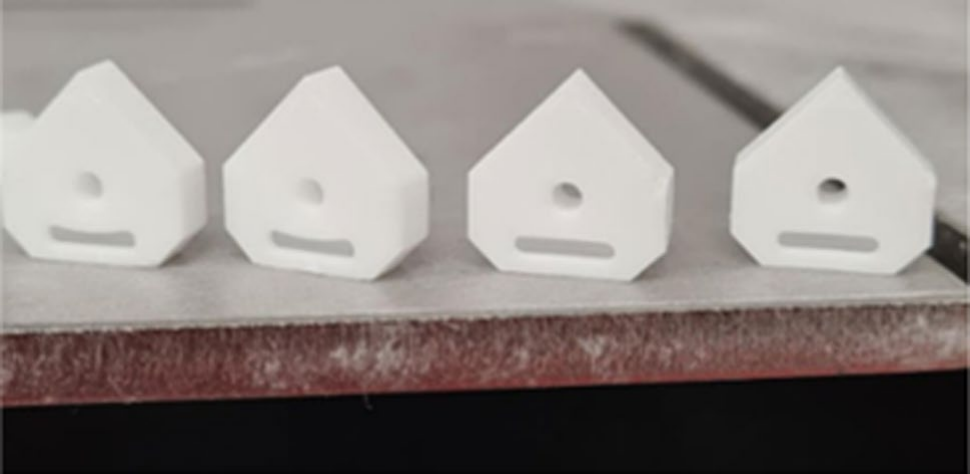
Sintered aluminum oxide inserts

On completion of the forming and cleaning phases, debinding was performed in a programable electric furnace (Carbolite Gero RHF 1600) followed by subsequent sintering in air using the parameters recommended by the manufacturer [34]. Figure 6 shows the green bodies and densified parts.

### Physical Prototype of the Tool Holder

**Fig. 7:**
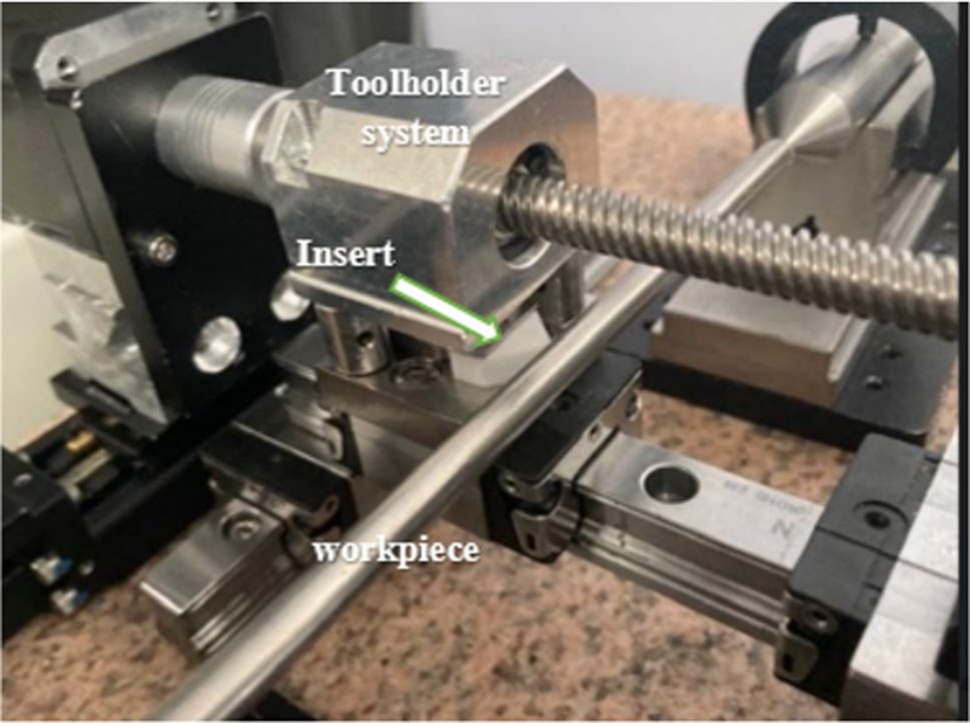
Image of the cutting insert relative to the workpiece

Figure 7 illustrates the mechanism of linear motion. This model shows how the toolholder is fixed rigidly in position atop a stainless-steel alloy. The cutting tool itself is then placed atop a predrilled threaded hole and locked in position. A stabilizing support is then bolted behind. The upper clamp, which contains the secondary neodymium magnets (as illustrated in Fig. 3b), is fixed over the cutting tool. Finally, optical posts are attached to a bearing unit, which provides the linear motion of the tool system controlled via the CNC drive unit. Table 3 presents the machining parameters and conditions.

**Table 3:** Machining conditions used for the experimental tests

Machining parameters	1	2	3
Cutting speed *V*_*c*_	250 m/min	500 m/min	900 m/min
Feed rate *f*	0.08 mm/rev	0.08 mm/rev	0.08 mm/rev
Depth of cut *a*_*p*_	0.1 mm	0.1 mm	0.1 mm

Machine setup	Description
Workpiece	6-mm AISI austenitic stainless steel 316
Cutting condition	Dry/internal cooling
Insert material	Al_2_O_3_
Insert geometric form	Customized
Turning machine	Customized

### Heat Sink

**Fig. 8:**
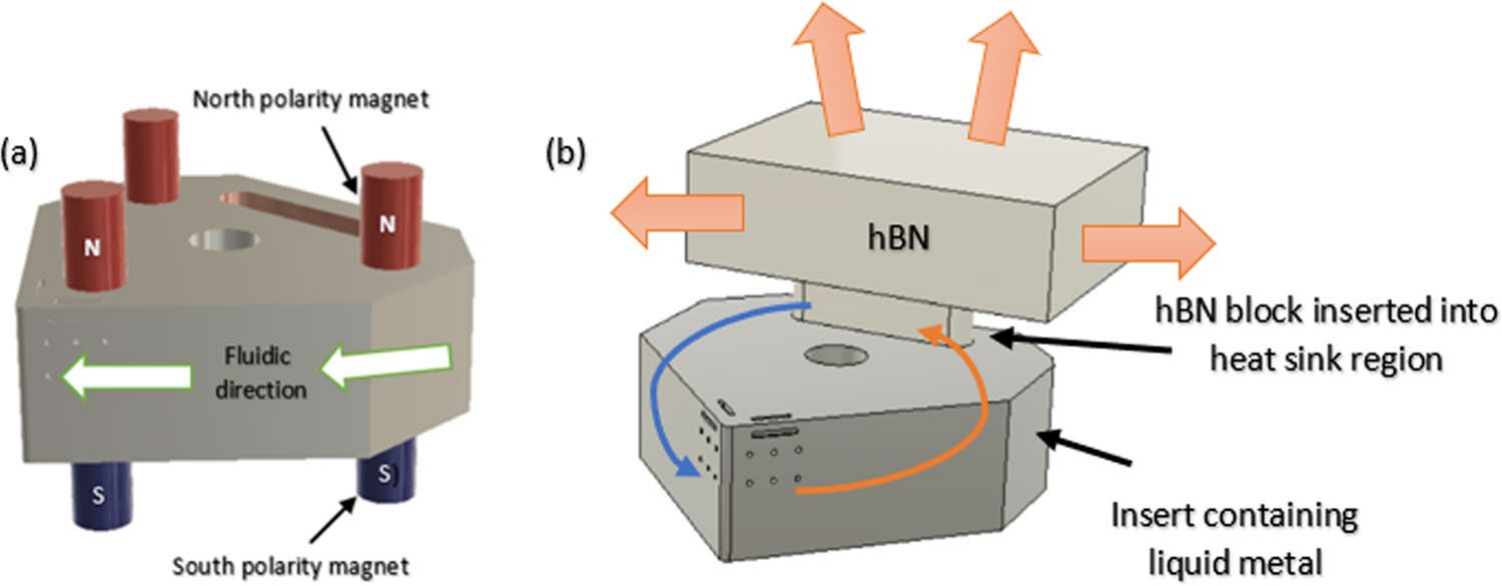
**a** Illustration of the MHD setup with neodymium magnets placed perpendicular to the liquid metal channel (magnets not to scale). **b** Mechanism of thermal transfer through the hBN heat exchanger

**Fig. 9:**
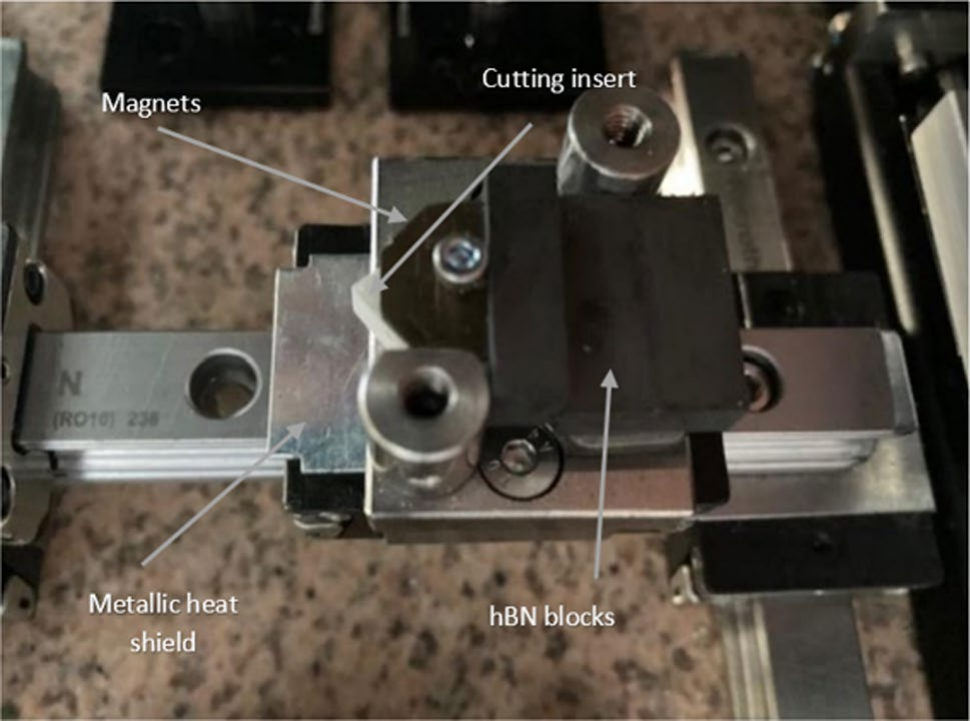
Images of the MHD-based coolant system mounted on a custom-made desktop-sized turning machine for the experimental testing of the tool. The hBN blocks are inserted into the heat sink region via the slot

The circulation of liquid gallium through the Lorentz force alone is insufficient to continuously transfer the heat generated by the interaction between the cutting tool and the rotating workpiece. The limitations are due to the finite heat capacity of the Al_2_O_3_ insert and the liquid metal within the small dimensions of the internal channel. This invariably introduces a limiting factor in the form of transient thermal equilibrium. Once the quantity of heat produced reaches a certain magnitude within the liquid metal, there will be a point at which the heat is no longer effectively removed as there is no temperature differential. To circumvent this, a heat sink is introduced in the MHD system (Fig. 8a, b). In addition to the suitability of the material in terms of its thermal conductivity, it is necessary to ensure that the selected material is compatible with liquid gallium in terms of wettability and corrosion resistance. Therefore, hot-pressed hBN was selected. A custom-made hot-pressed hBN part, which matches the geometry of the slot, was placed in position and locked in place. Figure 9 shows the physical prototype*.* The dimensions of the hBN heat sink (*L* × *W* × *H*) were 15 mm × 10 mm × 4.5 mm.

### Measurement of the Magnetic Field

**Fig. 10:**
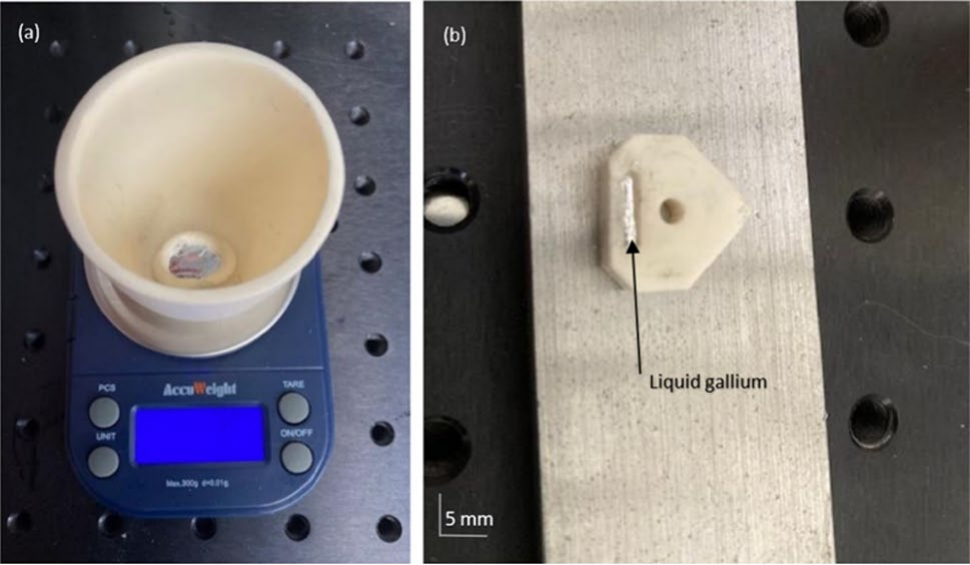
**a** Weighing of the liquid gallium in an alumina crucible. **b** Cutting insert containing liquid gallium to be used in the experiment

**Fig. 11:**
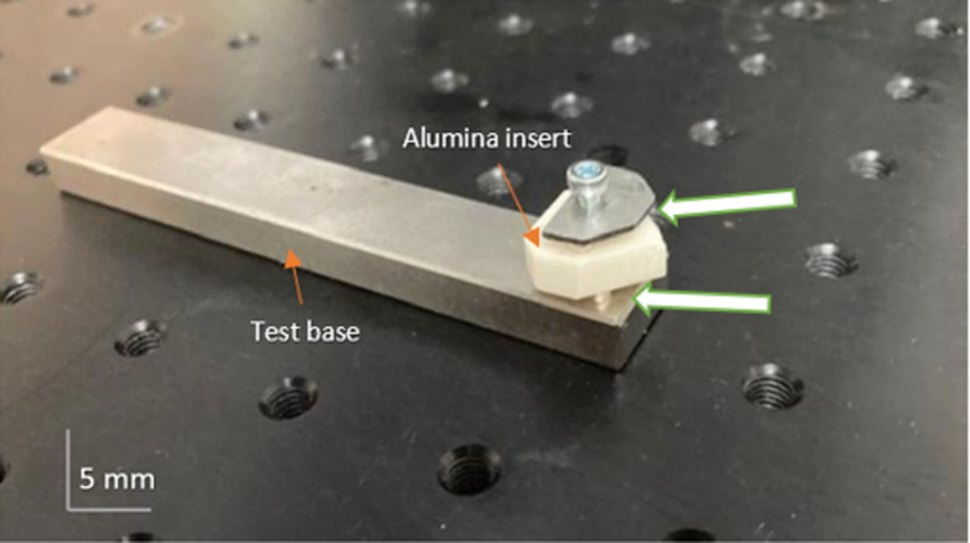
Experimental setup for magnetic field measurement. The arrows indicate the location of the neodymium magnets

Solid gallium (99.99% purity, Goodfellows®) was heated to temperatures above 29.8 °C using a conductive heat source until it reached a liquid state. It was then placed in an Al_2_O_3_ crucible, which was previously weighed (Fig. 10a), and the required volumetric amount was extracted using a coated syringe to ensure no wetting of the surface, which can lead to mass loss. The liquid gallium was then injected into the ceramic insert (Fig. 10b). The magnetic field within the internal channel in terms of the spatial distribution was measured using a Gaussmeter with a Hall-effect probe (Fig. 11). For the test, the Gaussmeter was fixed in a linear grip, which could be manipulated parallel to the direction of the liquid flow and thus provide a constant distance between the magnetic field lines and the sensor of the gauge.

To measure the applied magnetic field, a series of measurements were made using a magnetometer under a corresponding rise in temperature. Measurements of the initial magnetic field generated by the fixed neodymium magnets were taken as reference. The temperature rise was realized through a controllable burner with both the magnetometer and thermal imager fixed in the MHD direction (Fig. 11)*.*

### Thermographic Measurement of the Heat Transfer

The experiment was conducted using a set of controlled conditions. In this setup, liquid gallium was inserted into the open access port of the internal channel. Once inserted into the chamber, two electrodes located at the polar ends of the open access port created a constant current throughout the test. The heat was then applied from an initial temperature of 50 °C until a constant temperature of 600 °C was maintained at the edge of the Al_2_O_3_ tip. This heat range simulated the action of heat transfer in a typical cutting zone using the parameters for machining conditions. Table 4 presents the experimental conditions.

**Table 4:** Experimental conditions for the applied magnetic field

Parameter	Magnitude
Magnetic field intensity [mT]	0.98–1.2
Temperature of Al_2_O_3_ surface [°C] (before heating)	27.3
Temperature of Al_2_O_3_ surface [°C] (after applied heating)	124–367
Duration of applied heat [s]	30–60
DC current output [A]	25

**Fig. 12:**
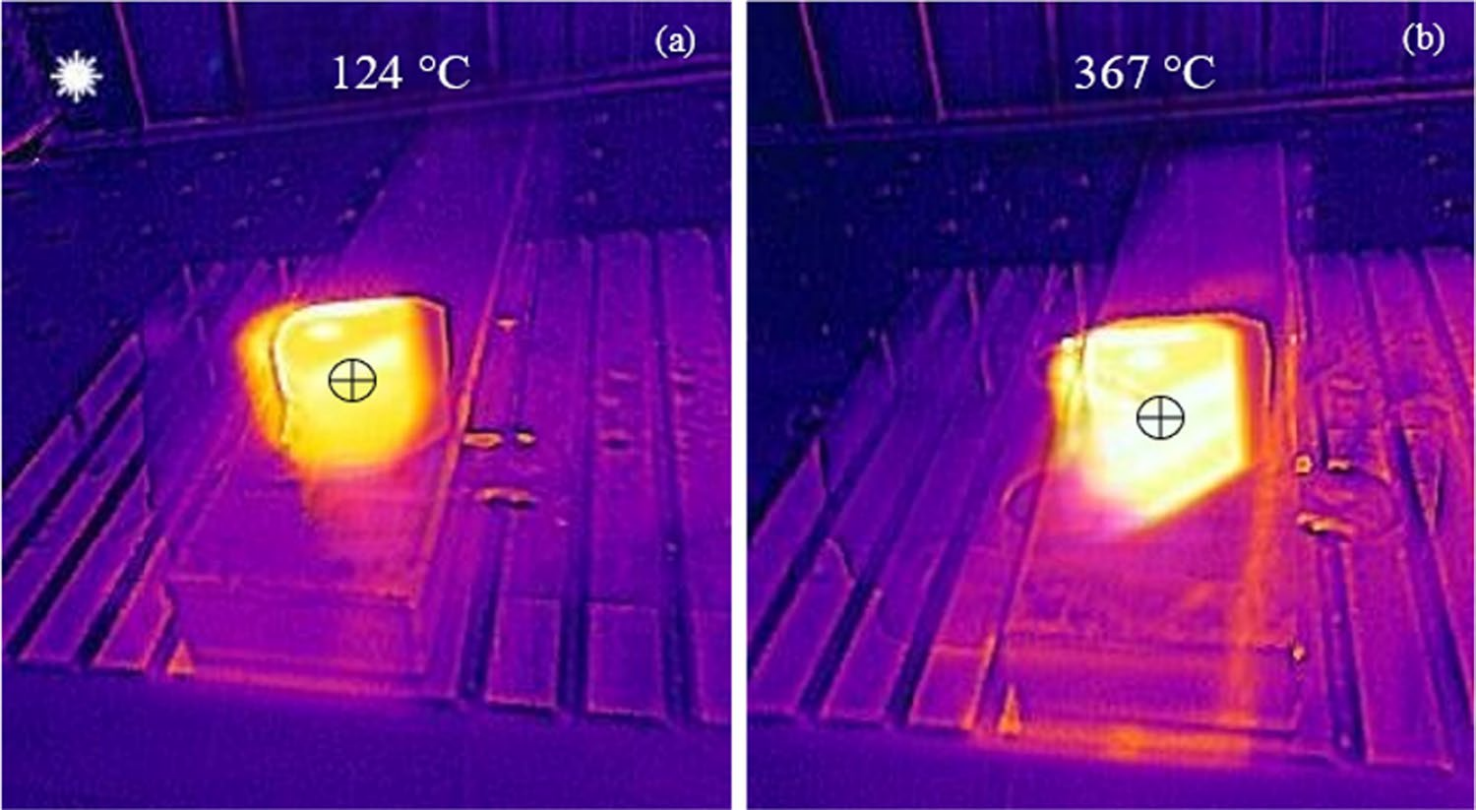
Thermographic measurements of the surface heat magnitude with liquid gallium in the internal channel after heating for **a** 30 s, **b** 60 s

A high-temperature thermographic camera (FLIR TG297) was used to capture the dynamic thermal variations on the insert surface during the period 30–60 s (Fig. 12a, b), in which a constant flame was applied at a fixed distance from the tool. A direct correlation between the rising temperature and the rate of heat transfer in the liquid could be observed. This can be understood from the higher thermal energy in the melt, producing a corresponding increase in the local velocity currents. The increase in the velocity of the liquid also had the effect of increasing the heat transfer rate. Therefore, the higher the applied temperature, the greater the ability of liquid gallium to remove heat from the internal structure of the tool. One of the challenges in measuring the magnetic field is preventing the heat from reaching the neodymium magnets. Permanent magnets are limited to operational temperatures below 80 °C, after which the magnetic field strength is reduced [27]. Therefore, a range of measurements was made to adjust for the variations in the magnetic excitation caused by exposing the permanent magnets to high temperatures. On average, the variation resulted in a magnetic field range of 0.94–1.2 T over 60 s. This is reflected in the numerical model with a magnetic flux density set to 1.05 T as opposed to the manufacturer rating of 1.2 T for the permanent magnets.

## Magnetohydrodynamic Simulation

### Model of the MHD System

To model the behavior of the liquid as it circulates through the channel, ANSYS^©^ Mechanical and FLUENT CFX code software tools were used for the numerical modeling. FLUENT, which is an FEA tool that uses an iterative solver program, was applied to obtain the numerical solution to the liquid gallium flow inside the cutting insert. A laminar flow model was employed through a Reynolds averaged Navier–Stokes simulation (RANS) model in the steady state.

For the MHD simulations, the electromotive force was added through an add-on module available through the FLUENT platform, in which the magnetic field induction equation was used. For the discretization of the domain, a volume mesh computed through the function tool of FLUENT was used. A numerical solution obtained using the ANSYS CFX solver provided the surface temperature fields for the rake, main flank face, and minor flank face. The approximation of the temperature field distribution was obtained using FEA at a fixed cutting depth and feed rate, with the cutting speed set to three settings, in conjunction with an internal cooling differential with liquid gallium as the medium. The dissipation and transfer of the generated heat is restricted to the tool, chip, and workpiece of the model. A conjugate heat transfer (CHT) model was used for the solid/liquid boundary interface of the internal channel. Tables 2, 4, and 5 present the parameters used for the model. The fluid parameters governing the MHD flow under the applied magnetic field have been defined in Sects. 2 and 3.

**Table 5:** Modeling parameters used in the thermomechanical analysis of the cutting insert

Thermomechanical	Magnitude
Heat flow	25 W
Temperature	
Rake edge	600 °C
Major flank edge	500 °C
Loading conditions	
Rake edge	700 N
Major flank edge	600 N

The model uses the geometric specifications of the Al_2_O_3_ insert containing the internal channel as defined in Appendix A. The internal channel contains liquid gallium, and the walls are considered to be electrically insulating, which is a good approximation for the properties of Al_2_O_3_.

**Fig. 13:**
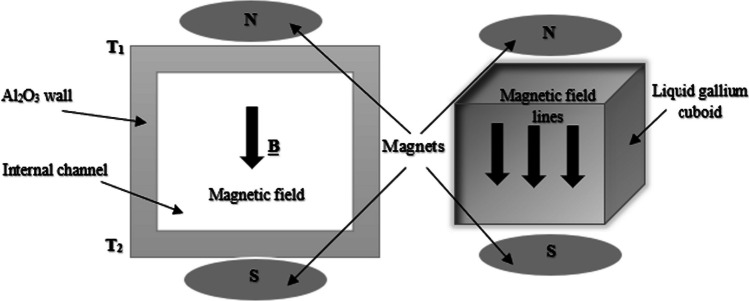
Diagram of the geometric and physical conditions of the internal channel with liquid gallium within the Al_2_O_3_ walls

The numerical model uses a geometrical configuration comprising a rectangular cavity representing the internal channel (Fig. 13), with the outer wall temperature *T*_1_ fixed at 600 °C and *T*_2_ at 500 °C. The temperature through the solid–liquid boundary is transient and depends on the thermal parameters defined in Tables 1, 2, and 4.

The simulation used in this study to approximate the solutions of the boundary conditions was solved under an induced magnetic field through a volume of the fluid solver. ANSYS Fluent with the add-on MHD function was employed in which the time-varying induction equation for the MHD system is given by the following:

5.1 $$\frac{\delta \mathrm{B}}{\delta t}+\left(U\cdot \nabla \right)B=\frac{1}{\mu \sigma }{\nabla }^{2}B+\left(B\cdot \nabla \right)U$$

Here, *B* is the magnetic field, *µ* is the magnetic permeability, and *σ* and *U* are the electrical conductivity and velocity of liquid gallium, respectively.

The boundary conditions are confined to the geometry of the insert and the internal channel within the solid–liquid boundaries of the material properties assigned to each condition.

To effectively assess the MHD system, it is necessary to integrate the fluid dynamic behavior under the influence of the applied magnetic field. In addition, the thermodynamic results are included in the model for completeness. This will help reflect the physical model, which uses all three parameters of influence under machining conditions. For the simulation, a thermomechanical model was imported directly from the ANSYS workbench into ANSYS Fluent. The MHD tool was then activated, allowing for the setup conditions and initialization of the MHD function. Because the configuration employs polar-directed magnets to generate the magnetic field, the direction of the applied field is fixed along the *z* direction in terms of the system orientation. Therefore, the magnitudes of the *x* and *y* fields were set to zero. Equation (5.1) is approximated through the program with the applied magnetic fields within the geometric dimensions defined by the CAD model. The properties of liquid gallium were imported from the FLUENT material database. However, in its elemental form, gallium does not have sufficient electrical conductivity to enable current density variation from the applied magnetic field. To replicate this in the model, the magnitude of the electrical conductivity was changed to mirror the effect of the DC current on gallium. To simplify the model, the magnetic field was made to act only on liquid gallium, which is a reasonable assumption, given that the ceramic insert is effectively electrically non-conductive. The variation in the magnetic pole distance is factored into the model to compensate for this omission. For the N35 grade neodymium magnets, an applied magnetic field in the range of 0.98–1.2 T was assumed, directed perpendicular to the liquid metal flow. This was the value measured using the magnetometer and was within the range specified by the manufacturer (Eclipse®). The solution iteration ended at 300 steps.

### Modeling Assumptions

To establish the numerical model of the liquid metal within the internal channel, the following assumptions were made:

- Fluid flow is contained within the dimensions of the internal channel.
- The fluid behavior is single phase and incompressible.
- No slip boundary occurs between the surrounding channel wall and the circulating fluid.
- The channel configuration uses a velocity inlet and pressure outlet.
- Gravitational and radiation effects are ignored.
- The applied magnetic field is perpendicular to the fluid region of the cutting tool only.
- The flow direction is horizontal to the applied magnetic field (*B*_*z*_), which is perpendicular to the liquid.
- Joule heating effects are ignored as the magnitude is low.
- The Lorentz force is the resultant interacting electromotive force on the velocity and momentum of the circulating liquid metal.
- The internal walls containing the liquid metal are electrically insulating and non-magnetic; therefore, all the electric currents end within the fluidic regions.

### Numerical Modeling and Analysis

Boundary conditions were applied to the solver only for the fluidic region. The external magnetic field *B*_*o*_ components for the magnetic induction were *x* = 0 T, *y* = 0 T, and *z* = 1.2 T. The solver was limited to the Lorentz force and MHD only, with Joule heating ignored. A transient condition was applied to the solver for a dynamic observation of the fluid behavior at *t* > 0. A viscous laminar behavior was initially applied to the model to reflect the expected velocity rate combined with the liquid metal properties. The simulation used a total of 63,795 nodes and 292,843 elements, with 15,797 nodes and 45,433 elements for the internal fluidic channel. For the simulation, the given values in Tables 2, 4, and 6 were used to reflect the physical properties of the material.

**Table 6:** Physical parameters used in the numerical model for liquid gallium

Parameter	Magnitude
Prandtl number (laminar)	0.028
Reynolds number	varies
Density [kg/m^3^]	5900
Thermal conductivity [W/m·K] (30 °C)	40
Specific heat [J/kg·K]	0.373 × 10^3^
Compressibility coefficient (at 20 °C)	2 × 10^−10^ m^3^/kg

To evaluate the effectiveness (or lack thereof) of liquid gallium as the heat transfer medium, we compared it with liquid water. For the liquid water model, the same conditions were used, albeit with the absence of the applied magnetic field, and modifications to its thermophysical properties were made.

## Simulation and Experimental Results

### Simulation Results

**Fig. 14:**
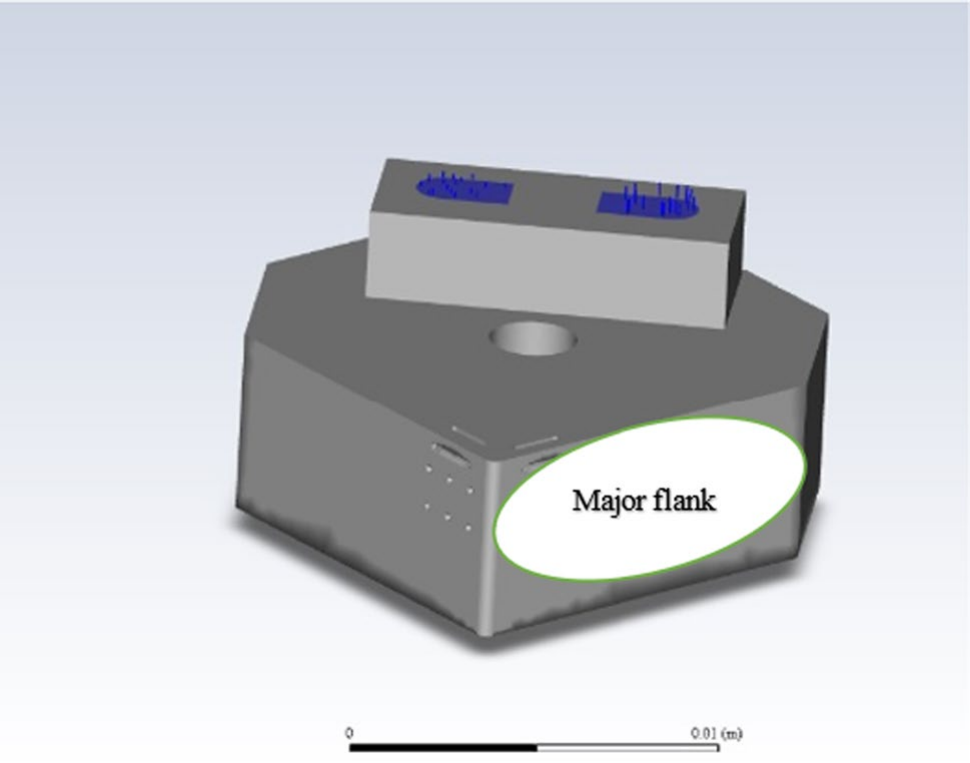
Illustration of the planar region on the major flank extracted from the CFD simulation

A plane section of the major flank (Fig. 14) was analyzed using the CFD post-processing tool. This was to ascertain the variations in the localized fluid velocity under the applied magnetic field and to measure the temperature contour distribution of liquid gallium in the internal channel.

**Fig. 15:**
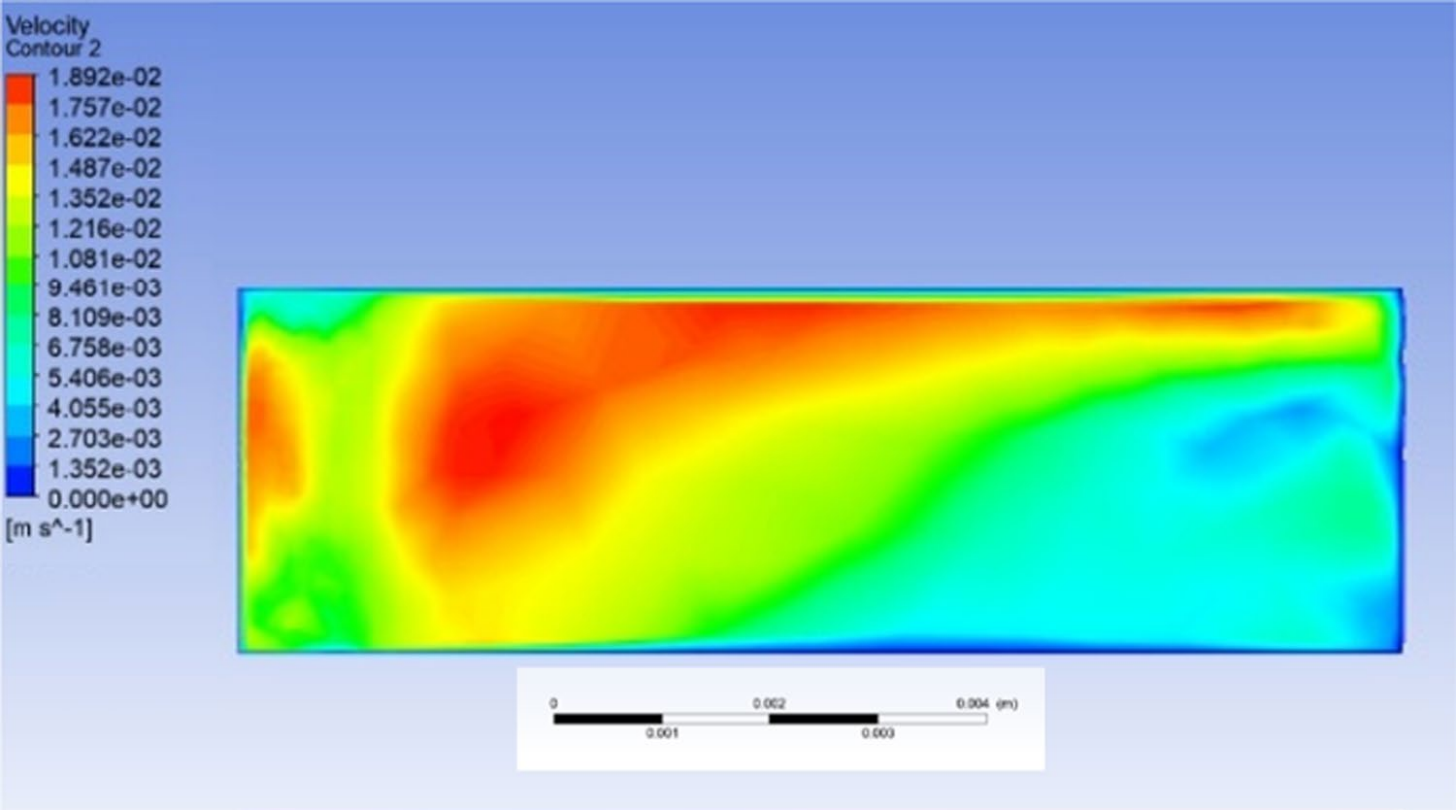
CFD velocity distribution simulation of liquid gallium flow under the effect of an external magnetic field. The plane section corresponding to the major flank region of the Al_2_O_3_ cutting insert

Figure 15 shows the CFD simulation of the 2D velocity distribution contour of the major flank within the internal channel*.* The image captured from the ANSYS FLUENT post-processing tool shows that liquid gallium exhibits a dynamic motion under the action of the applied magnetic field in the range of 1.0–1.2 T. The data show a maximum flow velocity of ~ 17.6 mm s^−1^ in the region near the upper section of the major flank relative to the proximity of the tool edge. The velocity distribution varies in magnitude in the region of the internal channel to the lowest value of ~ 3.1 mm s^−1^. The magnitude of this field corresponds to the actual field strength produced by the neodymium magnets located at the same physical distance from the internal channel. Therefore, the numerical model is accurate insofar as the geometrical configuration of the MHD cooling system is concerned. The flow rates increased as the magnetic field strength lines were strongest relative to the vectorial position of the permanent magnets, which were perpendicular to the fluid flow. This resulted in regions with the highest magnetic field strength, and the Lorentz force was also the highest, thus modifying the flow contours [35].

Figure 15 shows that the flow behavior is a dynamic process occurring in the central area of this region within the internal channel at the extracted major flank section. This is potentially due to the secondary flow resulting from the recirculation of the two end regions of the channel structure, attributed to the destabilization of the flow currents [36]. If this is the case, the end regions of the internal channel provide a means to recirculate liquid gallium, and the flow dynamics are altered by the applied magnetic field, where it becomes more uniform as time progresses over the length of the structure [36]. Combining this with the thermal currents generated from the heat influx, we find that heat is transferred within the internal channel. However, notably, as this is a dynamic process, the image extracted from the CFD post-processing tool represents an instant during the controlled time cycle. As such, it does not reflect the overall fluid velocity. In this case, the magnetic field is directed from the top of the insert (north polarity) to the bottom (south polarity), thus completing the homogeneous magnetic field lines. The Lorentz force is perpendicular to this field and governed by the right-hand rule. Therefore, if the case is reversed, i.e., if the direction of the polarity is inverted, the direction of the Lorentz force will also be reversed. This inversion of the magnetic field lines is not computed. Therefore, it is unclear if this would have a positive (increased velocity) or negative (reduced velocity) effect on the liquid distribution contours. It would be interesting to check whether this variable could modify the velocity profile, allowing for control of the heat transfer rate of liquid gallium.

### Heat Transfer in the CFD Model

**Fig. 16:**
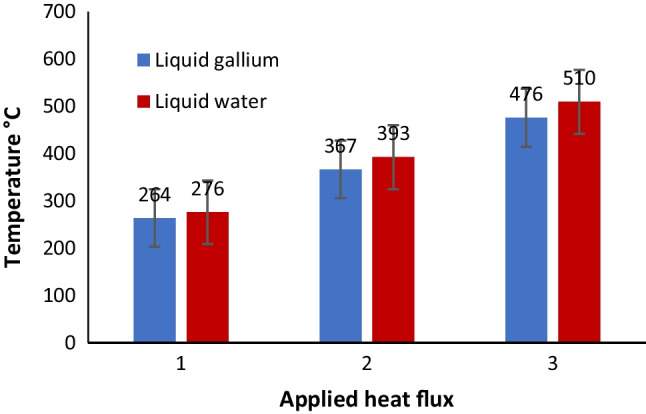
Block diagram of the varying temperature profiles at the cutting edge for liquid water and liquid gallium at three different cutting speeds: (1) *V*_*c*_ = 250 m min^−1^, (2) 500 m min^−1^, and (3) 900 m min.^−1^

Figure 16 shows a comparison of the resultant data for the CFD simulation of the two fluids. Liquid gallium outperformed liquid water in terms of heat transfer during all three applied heat flux conditions, which are reflective of the cutting speeds *V*_1_,* V*_2_, and* V*_3_. As indicated, both the cooling fluids were subjected to the same thermal conditions within the geometric dimensions of the insert and the applied boundary conditions contained within the developed numerical model. Furthermore, each simulation was conducted for the same number of cycles of the applied heat and at the same location in the cutting insert.

**Fig. 17:**
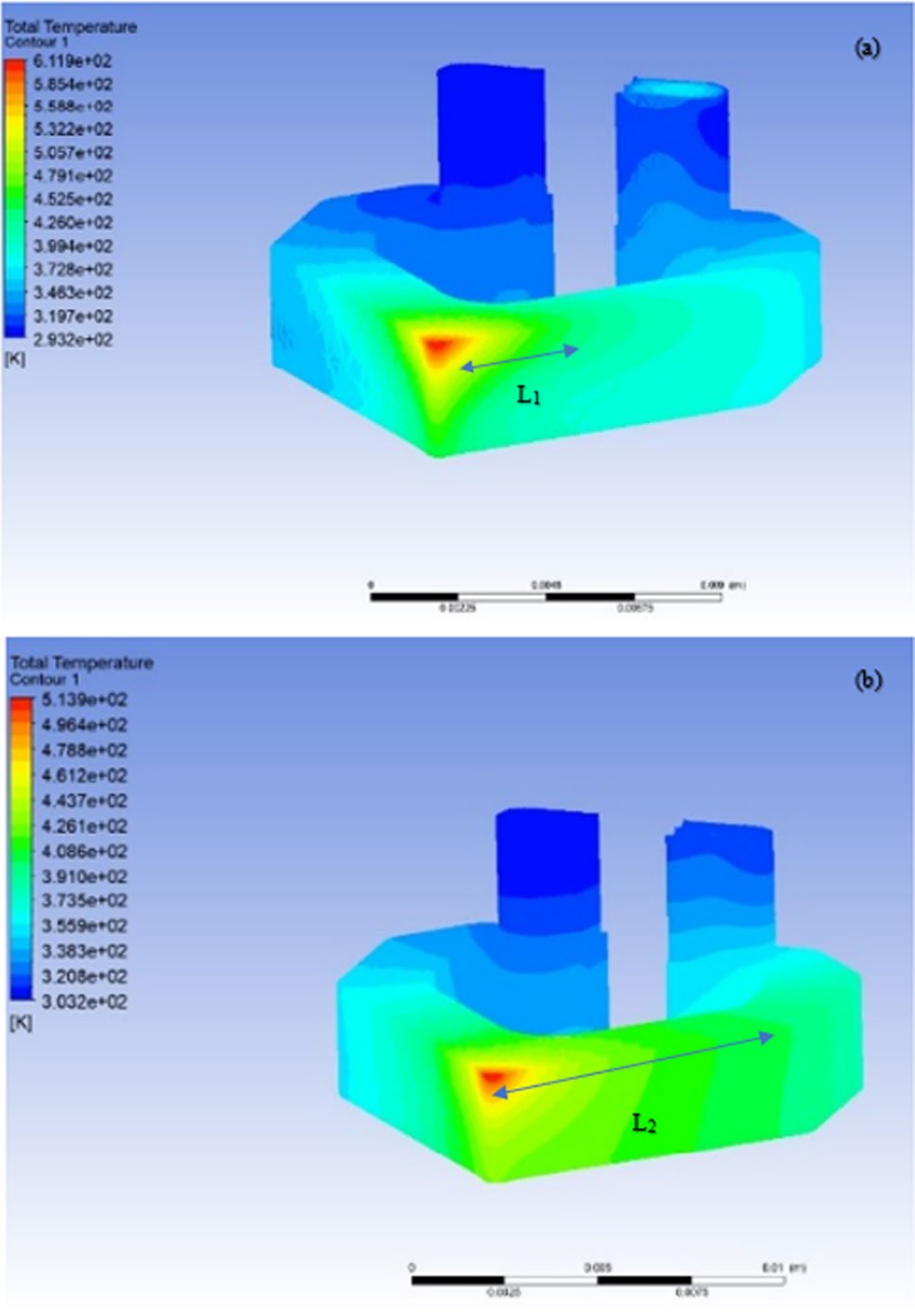
Thermal distribution contours for **a** liquid water and **b** liquid gallium on the internal channel surface of the Al_2_O_3_ insert at an applied temperature of 873.15 K (600 °C) on the solid surface

Figure 17 shows an extracted image from the CFD post-processing tool within the FLUENT program, showing the temperature distribution contour across the internal channel surface for liquid water and liquid gallium under the same applied heating conditions. A comparison of the two images shows that liquid gallium is more effective in transferring heat. Quantitatively, the difference at the peak cutting edge was 34 °C. Graphically, the images show how thermal transfer occurs. In Fig. 17a, the temperature contour color is reflective of the rate of heat transfer, which originates at the cutting edge and then moves across the major flank up to the heat sink. This is in contrast to that observed in the liquid gallium case shown in Fig. 17b. There is a wider heat plume distribution contour represented by the color change, showing how the thermal energy travels through the circulating fluid. In this case, the cutting-edge temperature is lower; however, the major flank region is at a higher temperature than that observed in the liquid water case. Furthermore, the higher heat plume extends further down the flank region toward the heat sink, which is graphically represented by L1 and L2 for liquid water and liquid gallium, respectively. This can be attributed to the better thermal transport ability of liquid gallium, which effectively carries the thermal currents away from the cutting edge and, in doing so, results in superior diffusion of the heat gradient.

### Heat Transfer Mechanism within the Internal Channel

**Fig. 18:**
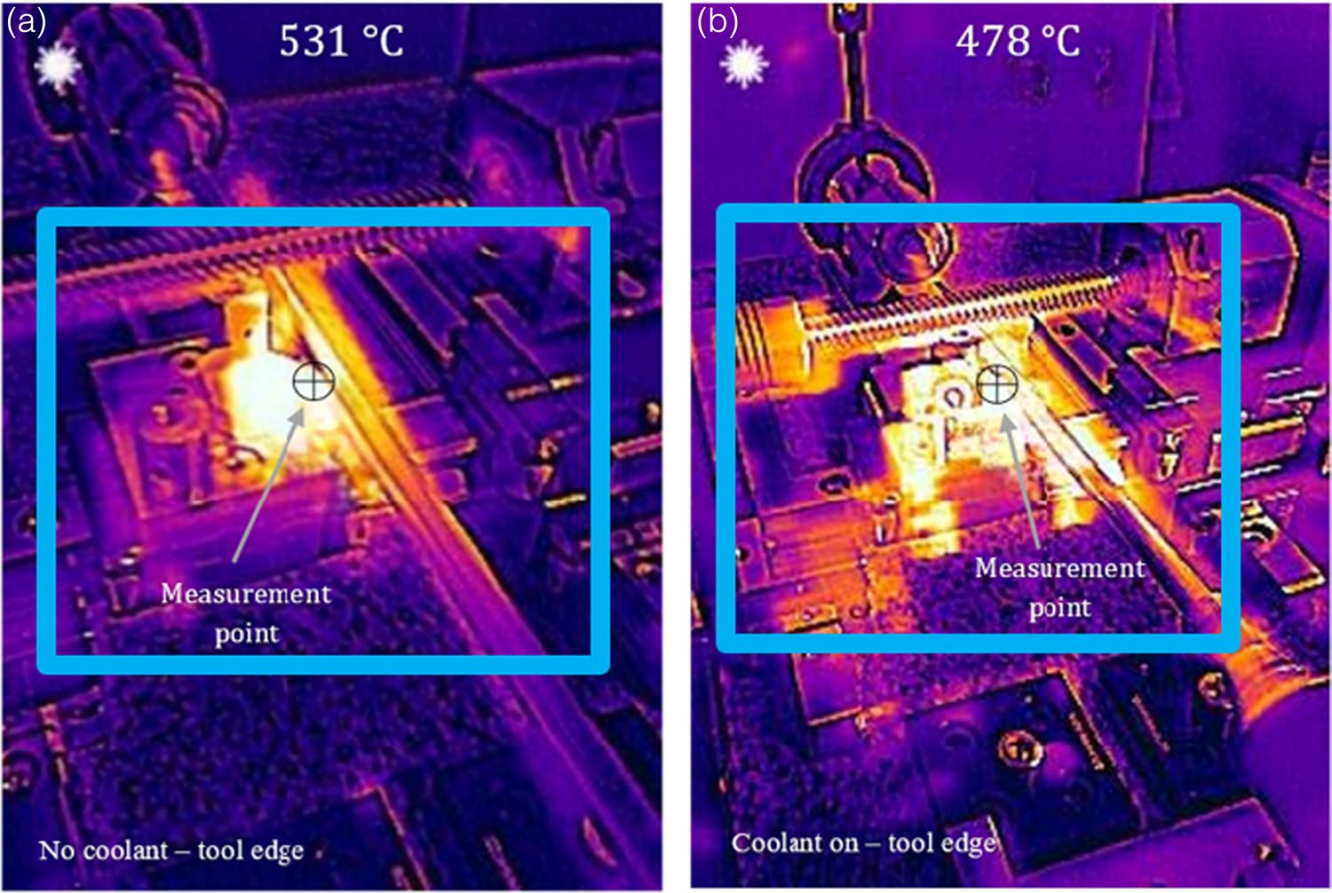
Thermographic images of the cutting insert, **a** with cooling off and **b** with the MHD cooling system on. The box shows regions with a difference in radiative heat transfer

The heat transfer mechanism can be further explained by examining the experimental machining tests using a thermographic imager. Figure 18 shows a higher dissipation of thermal energy within the liquid gallium, which correspondingly has a lighter hue reflective of the high heat (coolant on). The thermal image shows that the cutting-edge region has a temperature of 478 °C, with larger thermal plumes in the rear and flank areas of the insert with MHD coolant, as demonstrated by the brighter radiative image in this area. This was not observed in the insert when the coolant was not used, under dry conditions, recording a temperature of 531 °C (Fig. 18a, no coolant). Therefore, although the physical prototype does not use a variant internal coolant, i.e., liquid water, and as such, cannot be compared directly with the numerical model, the results indicate that the heat transfer increases over the solid surface of the MHD insert compared with the insert without cooling. The increase in the thermal energy across this region in the experimental test with the MHD system clearly shows that the heat is being transferred more effectively through the solid tool structure with the circulating liquid gallium, as opposed to that observed under dry machining conditions. This physical effect (heat transfer) is representative of the simulation model showing the heat distribution contour for circulating liquid gallium on the surface of the inner channel (Fig. 17b).

**Fig. 19:**
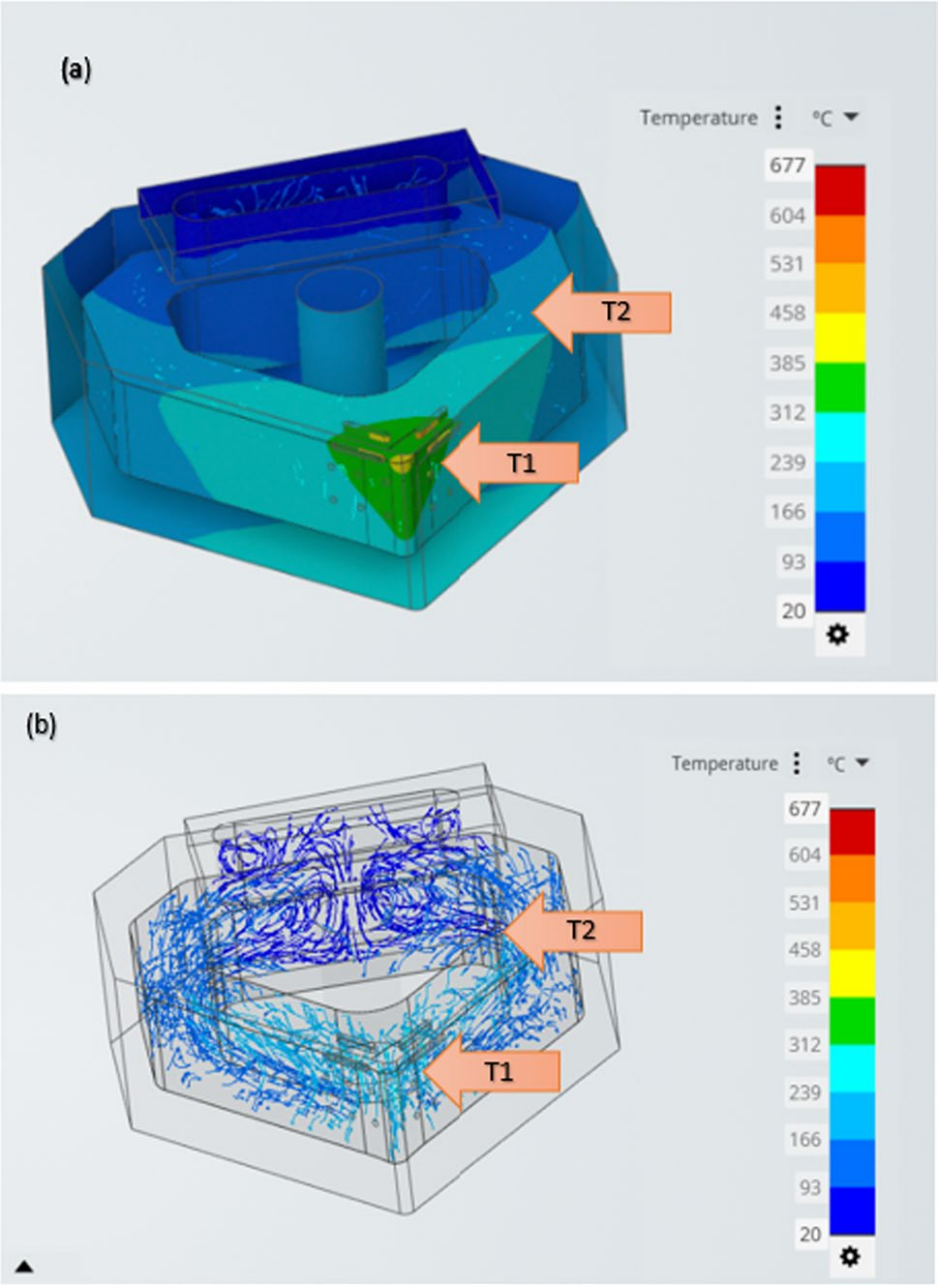
**a** CHT simulation of the heat transfer contours through the solid internal channel wall for liquid gallium. **b** Velocity vectors of the fluid within the internal channel at the same applied temperature of 600 °C. The arrows indicate the different temperatures in **a** the outer solid region and **b** inner fluidic region

Fluid dynamics of the thermal transfer can be seen from the simulation of the internal liquid flow, as shown in Fig. 19. When the heat reaches liquid gallium, convection currents increase, and they carry the thermal energy away from the primary heat source further into the channel. This effect is also visible in Fig. 17b. The velocity vectors indicate that the rise in the thermal energy increases the local fluid motion, thus removing the heat faster. As time progresses, the global velocity reaches a lower equilibrium concentration, and this then provides a conduit for the heat to flow. At this point, the circulating liquid gallium carries the thermal currents toward the heat sink and transfers energy to the hBN blocks. The higher viscosity of liquid gallium also contributes to maintaining the higher heat transfer rate compared with the use of liquid water (in the simulation model).

Figure 19a shows a CHT simulation of the heat transfer contours through the translucent border of the outer–inner insert solid. This example demonstrates the heat transfer of liquid gallium in motion through a time-lapsed image represented by T1 and T2. In Fig. 19b*,* the temperature difference between T1 and T2 represents the heat transfer in the inner fluidic region of the internal channel.

### Experimental Tests

Because of the configuration of the MHD cooling system and combined with the circulating liquid gallium, it was not feasible to construct a mirror test of the simulation experiments using liquid water as a comparative coolant substitute. Therefore, the obtained results could not be compared with the simulation model in terms of the internal liquid water coolant. Despite this, the resultant data were compared against those obtained under dry machining conditions in this first instance, with subsequent external cooling compared. This must be considered when evaluating the effectiveness of the MHD cooling system to provide a fair assessment of the actual magnitude of heat transfer relative to the model developed in this study.

Prior to the thermographic measurements, the thermal camera was calibrated to suitable emissivity levels for the ceramic material. As per initial tests, the workpiece was checked for tool run-out to reduce errors as much as possible. Three different cutting speeds were used in the trials while keeping the feed rate and depth of cut (DOC) constant (Table 3). The exact same parameters were used for the insert with the MHD cooling on and off. This was to ensure a fair measurement limited only to cutting-speed variation. The length of the workpiece engaged with the tool was 100 mm.

**Fig. 20:**
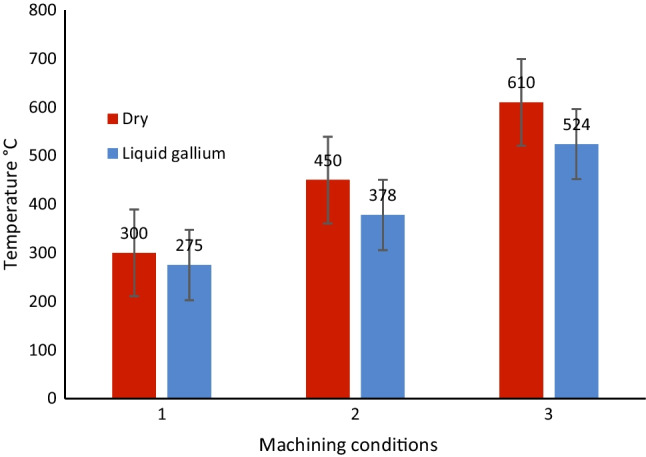
Block diagram of the varying temperature profiles at the cutting edge under dry conditions and with cooling on for three different cutting speeds. (1) *V*_*c*_ = 250 m min^−1^, (2) 500 m min^−1^, and (3) 900 m min^−1^

Figure 20 shows the resultant data of the varying temperature profiles at the cutting edge of the insert for all three cutting speeds. In all cases, the liquid gallium reduced the localized temperature in this region of the tool. Moreover, the reduction in the temperature increased with the increase in the cutting speed. This would suggest that gallium is either more effective at heat transfer because of the increased velocity currents and/or gallium becomes more thermally conductive with rising temperature. It is unclear if either of these explanations is conclusive. Nonetheless, as previously noted, the increase in heat affects the thermal properties of liquid gallium with respect to conductivity. Therefore, this might suggest a correlation between the data obtained at higher cutting speeds. Further investigations into the underlying mechanism(s) influencing this behavior would allow for enhanced control of the heat transfer and, in turn, superior thermal management at small scales.

### Tool Wear Results

One of the main causes of excessive tool wear is the high amount of thermal energy generated during uninterrupted cutting, such as turning operations. The optimization of suitable cutting parameters, cutting time, and tool wear rates is important for obtaining accurate measurements of surface roughness. However, our primary concern was the magnitude of the heat transfer in the MHD cooling system. Therefore, it was not within the scope of this work to include machining variables that can modify the resultant rate of tool wear or surface roughness. This would be prohibitively time-consuming and add complexity to solving what is effectively a heat transfer problem under controlled conditions. Therefore, the cutting time and cutting parameters (DOC and feed rate) were kept constant, apart from the cutting speed, as previously stated.

**Fig. 21:**
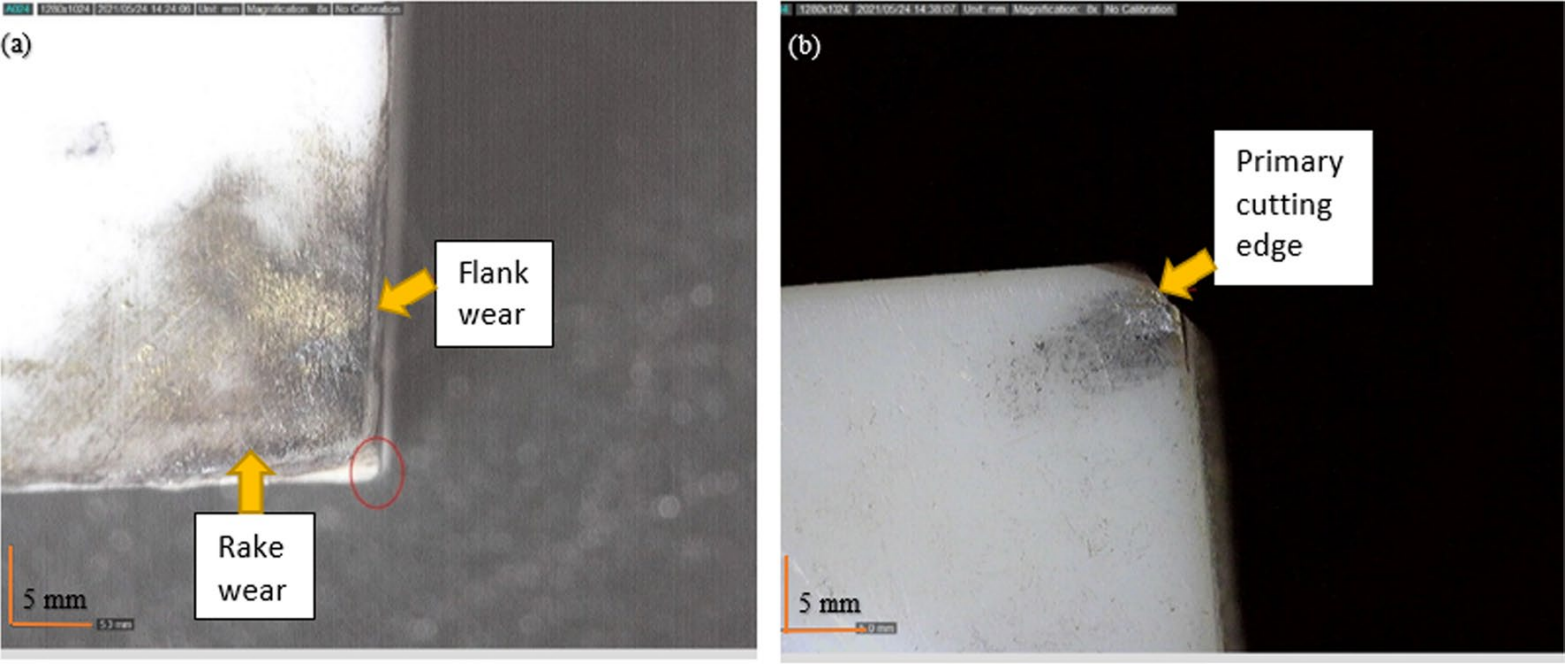
Optical images of the cutting insert exhibiting thermal wear ablation (**a**) without cooling and (**b**) with cooling

Although ceramics can provide good machining surface results when used in dry cutting operations, the excess heat generated can increase the tool wear rate, particularly in the rake and flank regions (Fig. 21a), in the form of thermal ablation. This is in contrast with the wear pattern shown in Fig. 21b under the same machining conditions with the MHD system being active. In this case, the thermal ablation could be reduced relative to the dry conditions.

**Fig. 22:**
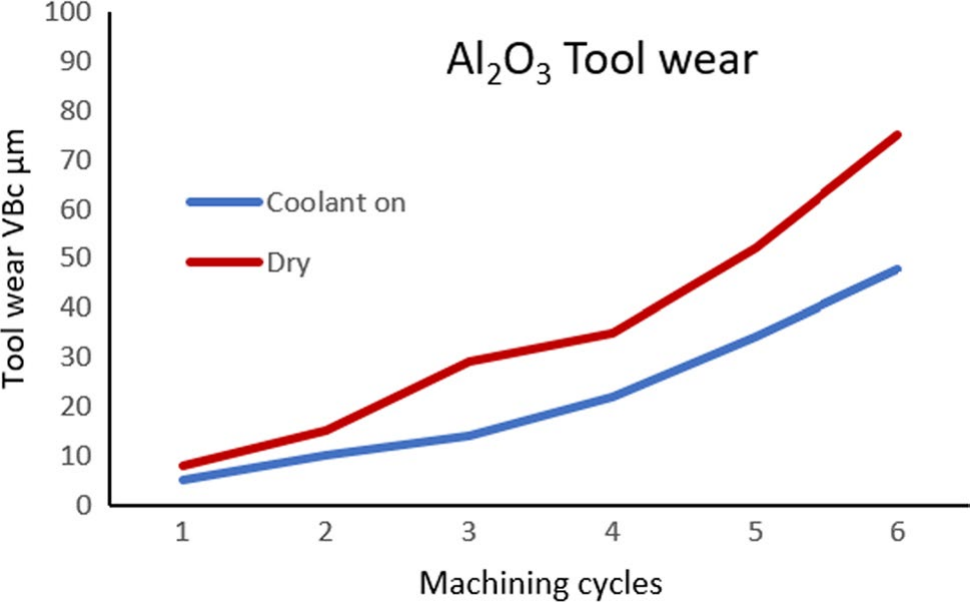
Corner wear VB_c_ rate corresponding to the number of machining cycles of the cutting insert under dry and cooling conditions at *V*_*c*_ = 250 m min^−1^

Figure 22 shows a graph of the corner wear rate VB_c_ relative to the number of machining cycles for the insert with cooling on and off for *V*_*c*_ = 250 m min^−1^. There is a difference in the wear rate VB_c_, and this difference increases with the increase in the number of cycles.

**Fig. 23:**
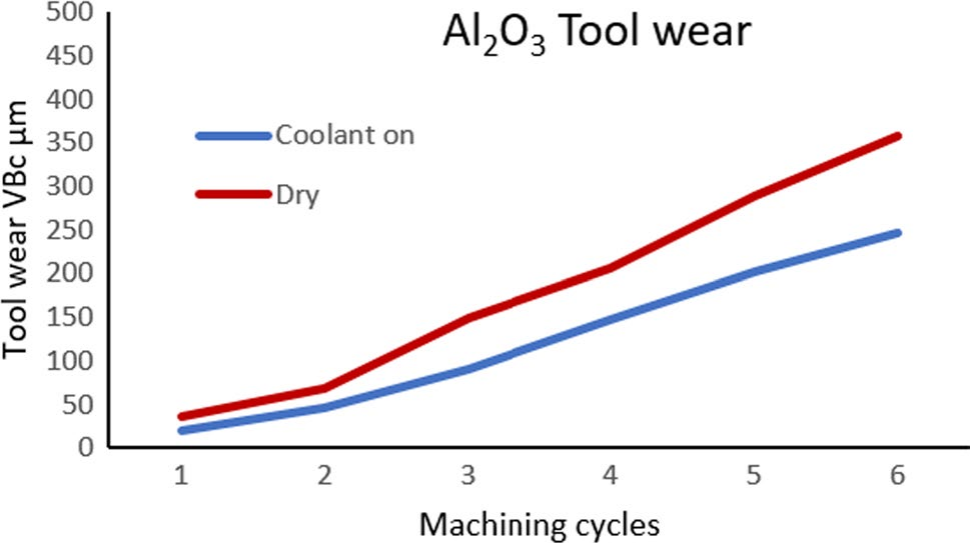
Corner wear VB_c_ rate corresponding to the number of machining cycles of the cutting insert under dry and cooling conditions at *V*_*c*_ = 900 m min^−1^

The effectiveness of the MHD is also clear at higher cutting speeds. Figure 23 shows the two corner wear rates VB_c_ corresponding to the number of machining cycles under dry and cooling conditions at *V*_*c*_ = 900 m min^−1^. Again, the insert with the MHD-based coolant outperforms in terms of the reduced wear profile after six cycles. This proves that liquid gallium could effectively transfer heat within the cutting insert during the experimental tests.

## Discussion

### Magnitude of Tool Wear Reduction

**Fig. 24:**
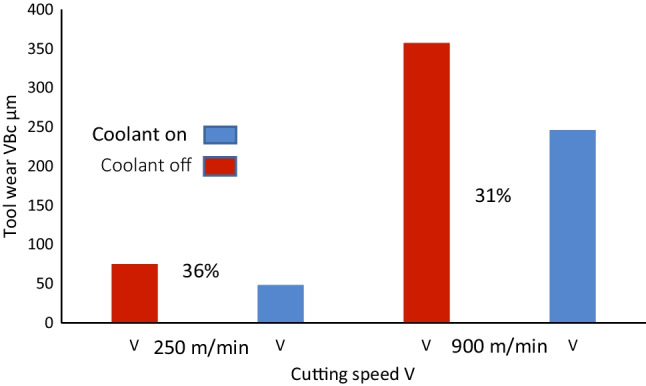
Tool wear reduction using the MHD cooling system. The graph illustrates tool wear conditions at two machining speeds, *V*_*c*_ = 250 m min^−1^ and *V*_*c*_ = 900 m min^−1^, with the corresponding percentage difference in the observed wear shown with the cooling on and off

Figure 24 shows the percentage of tool wear reduction at two cutting speeds over a period of six machining cycles. The results indicate that at a lower cutting speed *V*_*c*_ = 250 m min^−1^, the difference between the tool wear rate with the coolant being active relative to no coolant is 36%. When the cutting speed is increased to *V*_*c*_ = 900 m min^−1^, the difference between the tool wear rate with coolant being active relative to no coolant is 31%. Therefore, tool wear reduction is achieved using the MHD-based coolant. The results clearly indicate the potential of liquid gallium as a heat transfer agent in internal cooling applications and by extension demonstrable reduction in tool wear. Moreover, with the increase in the cutting speed, the generated heat increases, and the effectiveness of the heat transfer mechanism increases accordingly. This suggests that, at higher temperatures, the MHD cooling system removes thermal energy more efficiently.

### The Magnitude of Tool Wear Reduction: MHD Cooling Versus External Cooling

The reduction in tool wear is evident between the MHD cooling and dry cutting conditions. However, it can be asked to what percent this reduction translates when compared using the MHD cooling against liquid water as an external coolant. As noted previously, the configuration of the machine tool set up does not provide for liquid water to be pumped around the internal channel of the ceramic insert. As an alternative comparison, liquid water can soak the localized area of the cutting zone. This was done using a motorized pump which fed water through a malleable conduit at 2.5 mL s^−1^ at an ambient temperature of 20 °C.

**Fig. 25:**
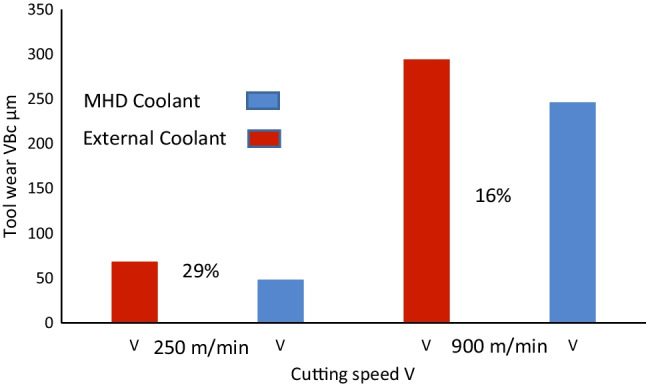
Tool wear reduction of the MHD cooling system vs external cooling. The graph illustrates tool wear conditions under two machining speeds *V*_*c*_ = 250 m min^−1^ and *V*_*c*_ = 900 m min^−1^, with the corresponding percentage difference in observational wear shown between liquid gallium (internal) and liquid water (external)

The resultant wear profiles of the two inserts are shown in Fig. 25. The cutting speeds are exactly as used in the previous conditions, namely, *V*_*c*_ = 250 m min^−1^, and *V*_*c*_ = 900 m min^−1^. At the lower cutting speed, the corner wear VB_c_ rate observed was 68 µm with the external coolant, and 48 µm with the MHD coolant. This represents a decrease of 29% in tool wear difference. When the cutting speed was increased to *V*_*c*_ = 900 m min^−1^, the corner wear VB_c_ rate showed 294 µm with the external coolant, and 246 µm with the MHD coolant. The difference between the tool wear rate reduction with the MHD coolant relative to the external coolant being 16%. Although this shows the internal coolant does transfer heat more efficiently than the external method, the following important points should be considered. (1) The cutting insert is monolithic Al_2_O_3_, as noted, water-based lubricants can impact wear rates, potentially resulting in dissolution of the Al_2_O_3_ microstructure under contact points [9]. Therefore, ceramic materials may not perform optimally when external coolants are applied. (2) It cannot be determined if this effect (the superior heat transfer and resultant reduction in tool wear) is limited to ceramic materials under these conditions. (3) Increasing the water flow rate may increase the heat reduction which in turn may improve the tool wear profile. Even if this is achievable, the fact remains that it would require larger volumes of water per second and risks potential fracture of the ceramic insert through thermal shock. Hence, there is a trade-off required corresponding to modification of the external method to compensate for the enhanced thermal transfer of the liquid gallium. In summary, the internal MHD cooing system is superior in terms of heat transfer compared to the external cooling method using liquid water, at a flooding rate common to low pressure fluidic coolants.

### Limitations of the Heat Sink

The hBN heat sink proved successful insofar as providing a means to maintain a temperature differential between the circulating thermal currents and the external air. Ultimately, however, the effectiveness of this method is limited by the temporal effect on thermal equilibrium rates within the system. To this end, it is envisaged that the heat, given sufficient time, would reach a steady state and effectively nullify the MHD heat transfer mechanism. However, the purpose of this study was to establish and subsequently demonstrate the validity of the design concept as a viable alternative to mechanically driven coolant systems. This also shows the potential of using closed cooling systems with liquid metals for enhanced heat transfer in internal cooling tools. The combined approach used in the numerical model through the integration of the thermal energy model within the MHD model demonstrates the usefulness of simulation tools to evaluate the effectiveness (or lack thereof) of the proposed system design.

### Liquid Gallium Versus Liquid Water

As noted in Sect. 6.2, it was not possible to experimentally compare liquid gallium against liquid water under the same conditions. This is due to the setup configuration of the MHD system. The prototype cutting insert does not use a mechanical input to pump the coolant around the internal channel of the insert. Therefore, replacing the liquid metal with liquid water would render the MHD system redundant. The MHD drive is a core aspect of the design; it overcomes the need for mechanical energy to facilitate the circulation of a fluidic substance. The permanent magnets, along with the resultant magnetic field, constitute a novel method to pump coolant around the defined geometry of the internal channel. It is not possible to remove this feature without changing the design parameters, which would affect the data obtained. Therefore, liquid water cannot be used within this design as a substitute for liquid gallium to measure the heat transfer difference within the internal channel.

Nevertheless, the simulation results indicate that liquid gallium can outperform liquid water under the same conditions. This gives confidence in the results obtained so long as this factor is observed. A modified design may enable a physical comparison of the two liquids under the same conditions in the future.

Considering the cost of gallium and the resulting magnitude of tool wear reduction, it is necessary to check whether the benefits outweigh the expense. The MHD system is reusable; the initial expense of gallium is effectively a one-off investment. Furthermore, in its elemental form, it can be reheated from a solid to a liquid when necessary, allowing for continuous use. Another factor to consider is the cost of the alternative: Mechanical input of liquid water requires a constant power source, which incurs additional costs. Therefore, although gallium is initially expensive, in the long term, it may provide a more sustainable solution to heat transfer in ceramic cutting tools if a superior form of heat sink can be integrated into the design.

## Conclusions

A numerical analysis of an MHD system design was conducted through a set of controlled parameters and boundary conditions to investigate the effects of the velocity characteristics of the liquid gallium cooling system within a ceramic cutting insert. A finite element analysis was performed based on the solutions of Maxwell’s equations coupled with Navier–Stokes equations governing the fluid flow behavior. The velocity of liquid gallium was found to increase under the action of a homogenous magnetic field applied by a neodymium magnet source. The source of the fluidic motion is the Lorentz force producing additional flow in the viscous liquid. The fluid dynamics obtained from the post-processing tool suggest that the behavior of liquid gallium is complex and, therefore, cannot be fully understood under the applied testing conditions. Further investigations are recommended in which the direction of the applied magnetic field can be varied. This may help confirm if there is any change in the velocity distribution relative to the current configuration of the north–south polarity in terms of the effectiveness of heat transfer.

The Lorentz force, which is generated due to the interaction between the magnetic field and a conducting liquid metal, can be engineered to modify the fluid behavior within the internal channel. Our numerical results showed that the flow rate increased through the action of the external magnetic field acting on the conducting liquid, producing a Lorentz force that caused additional electromotive flow in the MHD system. The experimental results showed that at a cutting speed of *V*_*c*_ = 250 m min^−1^, the corner wear VB_c_ rate was 75 µm with the coolant off and 48 µm with the MHD-based coolant on. At *V*_*c*_ = 900 m min^−1^, the corner wear VB_c_ rate was 357 µm with the coolant off and 246 µm with the MHD-based coolant on. These results represent a reduction of 36% and 31% in tool wear, respectively. When external cooling using liquid water was added, the results showed at *V*_*c*_ = 250 m min^−1^, the difference between the tool wear rate reduction with the internal coolant relative to the external coolant was 29%. When the cutting speed was increased to *V*_*c*_ = 900 m min^−1^, the difference observed between the internal liquid gallium coolant relative to the external coolant was 16%.

Furthermore, the results indicate that as the cutting speed increases, the liquid gallium provides increased heat transfer capabilities. This suggests a correlation between the increasing heat and the efficiency of liquid gallium as a thermal transfer agent. The study shows that MHD cooling with liquid gallium can serve as a potential approach for thermal management in ceramic cutting tools.

## Data Availability

The data that support the findings of this study are available from the corresponding author upon reasonable request.

## Appendix A: Geometry for Al_2_O_3_ Insert

**Table:**

Geometric Definition	Magnitude
Clearance angle major	0°
Insert angle	90°
Rake angle	0°
Cutting edge length	12.7 mm
Circle diameter	2.4 mm
Insert thickness	5.11 mm
Corner radius	0.3 mm
Cutting depth	0.01 mm
Internal channel (depth)	3.06 mm
Internal channel (width)	2.2 mm
Hemispherical surface patterning (diameter)	0.2 mm
Linear patterning (diameter)	0.2 mm
Linear patterning (width)	1.5 mm
